# Downregulating expression of OPTN elevates neuroinflammation via AIM2 inflammasome- and RIPK1-activating mechanisms in APP/PS1 transgenic mice

**DOI:** 10.1186/s12974-021-02327-4

**Published:** 2021-12-03

**Authors:** Long-Long Cao, Pei-Pei Guan, Shen-Qing Zhang, Yi Yang, Xue-Shi Huang, Pu Wang

**Affiliations:** College of Life and Health Sciences, Northeastern University, No. 3-11. Wenhua Road, Shenyang, 110819 People’s Republic of China

**Keywords:** Optineurin, Absent in melanoma 2, Receptor interacting serine/threonine kinase 1, Proteasome degradation, Alzheimer’s disease

## Abstract

**Background:**

Neuroinflammation is thought to be a cause of Alzheimer’s disease (AD), which is partly caused by inadequate mitophagy. As a receptor of mitophagy, we aimed to reveal the regulatory roles of optineurin (OPTN) on neuroinflammation in the pathogenesis of AD.

**Methods:**

BV2 cells and APP/PS1 transgenic (Tg) mice were used as in vitro and in vivo experimental models to determine the regulatory roles of OPTN in neuroinflammation of AD. Sophisticated molecular technologies including quantitative (q) RT-PCR, western blot, enzyme linked immunosorbent assay (ELISA), co-immunoprecipitation (Co-IP) and immunofluorescence (IF) were employed to reveal the inherent mechanisms.

**Results:**

As a consequence, key roles of OPTN in regulating neuroinflammation were identified by depressing the activity of absent in melanoma 2 (AIM2) inflammasomes and receptor interacting serine/threonine kinase 1 (RIPK1)-mediated NF-κB inflammatory mechanisms. In detail, we found that expression of OPTN was downregulated, which resulted in activation of AIM2 inflammasomes due to a deficiency in mitophagy in APP/PS1 Tg mice. By ectopic expression, OPTN blocks the effects of Aβ oligomer (Aβo) on activating AIM2 inflammasomes by inhibiting mRNA expression of AIM2 and apoptosis-associated speck-like protein containing a C-terminal caspase recruitment domain (ASC), leading to a reduction in the active form of caspase-1 and interleukin (IL)-1β in microglial cells. Moreover, RIPK1 was also found to be negatively regulated by OPTN via ubiquitin protease hydrolysis, resulting in the synthesis of IL-1β by activating the transcriptional activity of NF-κB in BV2 cells. As an E3 ligase, the UBAN domain of OPTN binds to the death domain (DD) of RIPK1 to facilitate its ubiquitination. Based on these observations, ectopically expressed OPTN in APP/PS1 Tg mice deactivated microglial cells and astrocytes via the AIM2 inflammasome and RIPK-dependent NF-κB pathways, leading to reduce neuroinflammation.

**Conclusions:**

These results suggest that OPTN can alleviate neuroinflammation through AIM2 and RIPK1 pathways, suggesting that OPTN deficiency may be a potential factor leading to the occurrence of AD.

**Supplementary Information:**

The online version contains supplementary material available at 10.1186/s12974-021-02327-4.

## Introduction

The pathological characteristics of Alzheimer’s disease (AD) are widely believed to be the deposition of extracellular β-amyloid protein (Aβ) and intracellular hyperphosphorylated tau, which is triggered by the impaired mitochondrial function [1–3]. Actually, the biogenesis of mitochondria was impaired via deactivating PGC1α-NRF-TFAM pathway in AD patients [4]. Furthermore, mitochondria dysfunction induces the production and deposition of Aβ in the brains of AD animals [5–7], suggesting the relationship between mitochondria impairment and AD. Reciprocally, the deposited Aβ in β-amyloid plaques (APs) may cause further mitochondrial dysfunction and impair mitochondrial biogenesis [8]. With regard to the mechanism, it is caused by the lacking of mitophagy, which is responsible for recycling and removing the impaired mitochondria via autophagy [9]. As a selective autophagy pathway, mitophagy deficiency was found to be related to AD by impairing synaptic function and memory [10, 11]. Moreover, activating mitophagy reduces the formation of APs and neurofibrillary tangles (NFTs) in AD patients and animals [10]. Except for AD, impaired mitochondria is recruited to mitophagy via a series of mitophagy receptors, such as the ubiquitin-binding receptors optineurin (OPTN) and p62 (SQSTM1) et al. [12]. Along these lines, mitophagy impairment has been suggested as a critical event in initiating AD, and restoring the function of mitophagy might be helpful for ameliorating the syndrome of AD [10, 13].

During this process, neuroinflammation seems to exert pivotal roles in accelerating the progression of AD. Consistent with this hypothesis, microglial cells have already been activated in the preclinical stage of AD as shown by positron emission tomography (PET) imaging [14]. In addition, inflammatory factors secreted by activated microglia can further exacerbate neuronal death [15]. In the brain of AD patients, many activated microglial cells have been observed around APs [16, 17]. This is ascribed to the existence of Aβ receptors, such as NOD-like receptors (NLRs) [18], Toll-like receptors (TLRs) [19] and receptor for advanced glycation end products (RAGE) [20], on the surface of microglial cells. Mechanistically, Aβ has the ability to penetrate the cell membrane of microglial cells to bind with the intracellular domains of NLRs, which activate NOD-, LRR- and pyrin domain-containing 3 (NLRP3) inflammasomes, leading to the release of proinflammatory cytokines, such as interleukin-1β (IL-1β), and resulting in neuronal death [21]. In cultured microglial cells, Aβ_1–42_ fibrils promote the maturation and secretion of tumor necrosis factor α (TNF-α) and IL-1β through TLR2, TLR4, TLR6 and CD36 receptors [22, 23]. In addition, RAGE can bind to Aβ to induce neurotoxicity by facilitating the secretion of IL-1β and TNF-α in microglial cells [24]. These findings all emphasize the important roles of neuroinflammation in AD.

Given the critical roles of neuroinflammation in AD, pyroptosis is a novel discovered mode of programmed cell death that is characterized by the rapid rupture of the cell membrane, leading to the release of proinflammatory factors. During the process of pyroptosis, cell lysis is primarily mediated by caspase-1 [25–27]. Caspase-1 activation has the ability to form pores of different sizes in the cell membrane [25], resulting in a reduction in the concentration of intracellular ions and an increase in osmotic pressure and cell swelling, leading to dissolution and release of inflammatory substances [28]. This observation is consistent with the phenomenon that the size of cells was increased when they were dying from pyroptosis [29]. This mechanism is supported by the fact that glycine specifically blocks ion flow in damaged eukaryotic cells, which prevents swelling and lysis during pinocytosis, acting as a cytoprotective agent [30]. Similar to this observation, the cytoskeleton is impaired in response to pyroptosis [31], even though caspase-1 is not involved in chromatin DNA cleavage during the course of pyroptosis [32].

Although pyroptosis mediates the effects of neuroinflammation on neuronal death, questions regarding the mechanisms by which cells sense intracellular and extracellular “danger” signals remain [33]. Among inflammatory pathways, TLRs can activate NF-κB, MAPK and interferon regulatory factors (IRFs), through which they induce the release of proinflammatory cytokines, such as NO, by activating microglia [34]. Similar to TLR4, the signaling cascades of inflammasomes are activated when NLRs nucleotide-binding oligomerization domain-containing protein 1 (NOD1) and NOD2 are recognized and activated by their ligands, leading to the release of proinflammatory cytokines, such as IL-1β and IL-18, through which pyroptosis occurs [35]. In addition, both TLRs and NODs can induce the production and accumulation of pro-IL-1β in microglial cells. Moreover, activation of TLR2 or TLR4 alone can promote the maturation and secretion of IL-1β, which is mediated by stimulating caspase-1 and accelerating the release of endogenous adenosine triphosphate [36]. Due to the central roles of caspase-1 and IL-1β in inflammasomes, all of this evidence indicates the involvement of TLRs and NODs in activating inflammasomes.

The inflammasome is composed of multiple cytoplasmic proteins, including NOD-like receptors (NLRs) or absent in melanoma 2 (AIM2), caspase-1 and apoptosis-associated speck-like protein containing a C-terminal caspase recruitment domain (ASC), which activate and maintain immunity under both physiological and pathological conditions [37]. Notably, ASC is responsible for recruiting NLRs to caspase-1 [38]. Based on their different N-terminal domains, the NLR family can be divided into several subfamilies, such as NLRP and AIM2. For NLRP subfamily proteins, the N-terminus contains a pyrin domain (PYD), which interacts with the PYD domain of ASC, leading to the formation of a complex with pro-caspase-1. Meanwhile, NLRPs contain a caspase recruitment domain (CARD), which can directly bind to pro-caspase-1 [39]. AIM2 inflammasomes can bind to DNA through their HIN200 domain, which mediates ASC oligomerization to initiate the activation of caspase-1-dependent inflammatory bodies, leading to the maturation and secretion of the proinflammatory cytokines IL-1β and IL-18 [40].

During the activation of inflammasomes, TLRs mediate the effects of ligands or endogenous stimulators, such as Myd88 and TRIF, on the activation of NF-κB or AP-1, resulting in upregulation of NLRP3 expression [41]. By activating NLRP3, large protein complexes of inflammatory bodies are formed by recruiting ASC and caspase-1 into the inflammasomes, leading to the hydrolytic activity of caspase-1, which results in cleavage of pro-IL-1β and pro-IL-18 to produce mature and bioactive forms of IL-1β and IL-18 [37]. In addition to this canonical inflammasome-activating pathway, lipopolysaccharide (LPS) has the ability to bind and activate caspase-11 [37], which results in the aggregation of NLRP3, leading to accelerated maturation and secretion of IL-1β and IL-18 [42]. Activated caspase-11 can also induce cleavage of the N-terminal region of gasdermin-D (GSDMD), leading to pyroptosis [41]. As the natural ligand of TLR4, LPS has been proposed to activate the NLRP3 inflammasome via Toll-like receptor adaptor molecule 1 (TICAM1/TRIF)-, receptor interacting serine/threonine kinase 1 (RIPK1)-, FAS-associated death domain protein (FADD)- and caspase-8-activating pathways [36, 43]. In addition to NLRP3 inflammasomes, AIM2 inflammasomes have the ability to activate signaling cascades of cGAS-STING-TBK1-NF-κB, leading to the synthesis of proinflammatory cytokines, such as IFN-β and IL-1β [44].

In AD transgenic mice, activation of the NLRP3 inflammasome promotes the production and deposition of Aβ, and knocking out expression of NLRP3 improves spatial memory by reducing the deposition of Aβ in the brain [45]. Reciprocally, the deposition of Aβ in microglial cells activates NLRP3 inflammasomes, leading to the maturation and secretion of IL-1β [21], which accelerates AD progression. Due to the interaction between RIPK1 and the NLRP3 inflammasome [46], RIPK1 is likely involved in regulating the pathogenesis of AD via inflammatory mechanisms. Indeed, mRNA and protein expression of RIPK1 is markedly increased in AD patients compared to control subjects [47]. Since RIPK1 is highly expressed in microglial cells in mouse and human brains [48], it is generally believed that RIPK1 plays an important role in neuroinflammation [49]. Inhibiting the kinase activity of RIPK1 induces microglia microglial cells to degrade Aβ [48].

Based on these clues, we identified OPTN for the first time as an essential receptor for mitophagy that regulates neuroinflammation. Specifically, OPTN deficiency in AD activates AIM2 inflammasomes and RIPK1-mediated inflammation. Furthermore, OPTN expression blocks the activation of AIM2 inflammasomes by inhibiting the expression of AIM2, ASC, caspase-1 and IL-1β in microglial cells. In addition, OPTN suppresses the translocation of NF-κB from the cytosol to the nucleus by inducing ubiquitin-dependent RIPK1 degradation mechanisms. Through these mechanisms, OPTN overexpression suppresses neuroinflammation during the course of AD development and progression.

## Materials and methods

### Reagents

Horseradish peroxidase-labeled secondary antibodies were purchased from Sigma-Aldrich (St. Louis, MO, USA). Antibody specific for OPTN (sc-166576, mouse, 1:1000 for WB) were obtained from Santa Cruz Biotechnology. Antibodies against AIM2 (sc-293174, mouse, 1:500 for WB), ASC (sc-514414, mouse, 1:500 for WB) were obtained from Santa Cruz Biotechnology (Santa Cruz, CA, USA). Fluorescence-tagged secondary antibodies (A32732 rabbit, A11034 rabbit, A32727 mouse, A11029 mouse, 1:500 for IF) were purchased from Thermo Fisher Scientific (Waltham, MA, USA). Other antibodies, including β-actin (#3700, mouse, 1:2,000 for WB), Histone (#4499, rabbit, 1:2000 for WB), IL-1β (#12242, mouse, 1:2000 for WB), RIPK1 (#3493, rabbit, 1:2000 for WB), p-IKBα (#2859, rabbit, 1:2000 for WB), IKBα (#4814, mouse, 1:2000 for WB), GFAP (#80788, rabbit, 1:5000 for WB), ubiquitin (#3933, rabbit, 1:1000 for WB) were from Cell Signaling Technology (Danvers, MA, USA). An Iba1 antibody (rabbit, 1:200 for IHC) was purchased from Wako Life Sciences (Wako, Tokyo, Japan). Antibody against caspase-1 (22915-1-AP, rabbit, 1:1000 for WB) and Flag (66008-3-Ig, mouse, 0.5–4.0 ug for IP and 1:2000 for WB), and HA (51064-2-AP, rabbit, 0.5–4.0 ug for IP and 1:4000 for WB) were purchased from Proteintech (Wuhan, Hubei, P.R.C). All reagents for the sodium dodecyl sulfate polyacrylamide gel electrophoresis (SDS-PAGE) experiments were purchased from Bio-Rad Laboratories (Hercules, CA, USA). Antibody specific against NF-κB (ab16502, rabbit, 1:2000 for WB) and HSP60 (ab190828, rabbit, 1:1000 for WB) were obtained from Abcam (Cambridge, MA, USA). DAPI was obtained from Beyotime Institute of Biotechnology (Haimen, Jiangsu, China). Bafilomycin A1 (Baf A1) was obtained from MedChemExpress (Monmouth Junction, NJ, USA). High-fidelity restriction enzymes for XmaI, SalI, XhoI, and HindIII were obtained from New England Biolabs (Beverly, MA, USA). All reagents for the quantitative real-time PCR (qRT-PCR) experiments were purchased from Bio-Rad Laboratories (Hercules, CA, USA). All other reagents were from ThermoFisher Scientific (Waltham, MA, USA) unless specified otherwise.

### Tg mice and treatments

APP/PS1 (Stock No. 004462) were obtained from the Jackson Laboratory (Bar Harbor, ME, USA). Wild-type (WT) mice were purchased from Liaoning Changsheng Biotechnology Co., Ltd. (Benxi, Liaoning, China). The neurons in the brains of APP/PS1 Tg mice doubly expressed a chimeric mouse/human amyloid precursor protein (Mo/HuAPP695swe) and a mutant human PS1 (PS1-dE9). Both mutations are associated with the early onset of AD. Tg mice showed deposition of Aβ at approximately 6–7 months of age. At 9 months of age, APP/PS1 Tg mice showed obvious impairment of learning ability compared with that observed in WT mice. Five mice per cage were housed in a controlled environment at standard room temperature and relative humidity with a 12-h light–dark cycle and free access to food and water. The general health and body weights of the animals were monitored daily. The brains of animals in different groups were collected under anesthesia. Subsequently, the brains were fixed through perfusion, as previously described [50].

### Aβ_42_ oligomer preparation

Aβo was generated as in previous study [51]. β-amyloid_42_ was purchased from ChinaPeptides Co., Ltd. (Shanghai, China). Specifically, lyophilized Aβ_42_ were dissolved in 1,1,1,3,3,3-hexafluoro2-propanol (HFIP; Sigma-Aldrich St. Louis, MO, USA) to 1 mM. The solution was bisected, the HFIP was evaporated, and the peptide was stored at − 80 °C. 24 h prior to use, amyloid peptide was dissolved (100 M) in dimethyl sulfoxide (DMSO; Sigma-Aldrich St. Louis, MO, USA) and ultrasound treatment. The solution was diluted to 20 M in F12/DMEM (glutamate-free, Kibbutz Beit-Haemek, Israel) and incubated at 4 °C for 24 h to obtain the Aβ oligomer (Aβo). The quality of oligomers product was controlled by Western blot using against Aβ peptide antibody (Sigma-Aldrich St. Louis, MO, USA).

### Cell culture

Mouse BV2 cells were grown (37 °C and 5% CO_2_) on 6 cm tissue culture dishes (1 × 10^6^ cells per dish) in appropriate medium. In a separate set of experiments, the cells were grown in serum-free medium for an additional 24 h before incubation with inhibitors in the absence or presence of Aβo [52].

### Culture of primary microglia cells

The plates were incubated with 0.01% l-polylysine at 37 °C for 4 h. Remove the l-polylysine solution, rinse the culture dish with sterilized deionized water, and dry it for standby. The mice born within 24 h were disinfected with 75% (volume fraction) alcohol. The mice were decapitated under aseptic conditions. The brain tissue was taken out and placed in a cold plate of pH 7.2, d-hank’s solution without calcium and magnesium (with ice bag under it). The cerebellum, hippocampus and cerebral medulla were removed under aseptic conditions, and then the cerebral cortex was obtained. The meninges and blood vessels were carefully stripped. The tissue was cut into 1 mm^3^ tissue pieces with iris scissors, and then digested with 0.125% trypsin and DNA enzyme at 37 °C for 20 min, and shaking for 2–3 times. Discard the supernatant, add complete inoculation solution to stop digestion, rinse twice. After resuspension with complete medium, let it stand for 2 min. Then the suspension was carefully collected in a new centrifuge tube, centrifuged (1000 rpm/min, 10 min, 4 °C), and the supernatant was discarded. Add the complete culture medium, and then resuspend and filter with 200 mesh stainless steel mesh. The cell filtrate was inoculated into the culture dish. After 24 h of incubation in 5% CO_2_ incubator at 37 °C, the medium was changed, and then the medium was changed every 3 days. The cells were cultured for 14–16 days. The culture medium was poured out and digested with 0.05% trypsin (2–3 ml). When the microglia attached to astrocytes were detached, the digestion medium containing floating microglia was transferred into a 10 ml centrifuge tube, and the digestion was immediately terminated with complete medium. 1000 rpm/min, centrifugation for 5 min, discard the supernatant, add complete culture, blow into cell suspension again, inoculate in the coated culture dish, and place in CO_2_ constant temperature cell incubator (37 °C). After 24 h, the culture medium was sucked out to remove the adherent oligodendrocytes, and the complete culture medium was added to continue the culture.

### Acquisition of fluorescence images

Cells grown on gelatin-coated coverslips, which were co-transfected with green fluorescent protein-OPTN (GFP-OPTN) and RFP-RIPK1 or pCHAC-mt-mkeima, respectively. After 48 h, the cells were fixed with 4% paraformaldehyde for 10 min, permeabilized with 0.1% Triton X-100 for 10 min, washed three times with phosphate-buffered saline PBS (−), and stained with DAPI. Images were captured and processed using a confocal microscope system obtained from Leica (Wetzlar, Germany), equipped with a 63 × 1.4 numerical aperture oil differential interference contrast Plan-Apochromat objective at room temperature.

### Co-immunoprecipitation (CoIP)

Transfected 293T or infected BV2 cells were lysed in lysis buffer (50 mM Tris–hydrochloride, pH 7.4, 150 mM sodium chloride [NaCl], 1% Nonidet P-40, 0.25% sodium deoxycholate, and 1 mM ethylenediaminetetraacetic acid, with protease and phosphatase inhibitors) for 1 h. The lysed cells were then centrifuged to remove cell debris. Protein amounts were quantified using a bicinchoninic acid protein assay kit (Thermo Fisher Scientific, Waltham, MA, USA). A fraction (1/10) of the cell lysate supernatant was heated as whole-cell lysate for immunoblotting. Co-immunoprecipitation was performed by incubating the remaining lysate supernatant (9/10) with 1 mg of the indicated antibodies or control IgG overnight at 4 °C for 16 h. The immune complexes were captured using Dynabeads obtained from Thermo Fisher Scientific (Waltham, MA, USA) for 3 h at 4 °C, under gentle shaking. The immunoprecipitates were washed five times, eluted by addition of sample buffer, boiled, and analyzed through SDS-PAGE. For ubiquitination, cells were initially lysed with radioimmunoprecipitation assay (RIPA) buffer containing 1% SDS. Subsequently, the cell extracts were diluted with RIPA buffer to 0.1% SDS. Finally, a fraction of the diluted extracts (1/10) was heated as whole-cell lysate for immunoblotting and the remained lysate (9/10) was subjected to immunoprecipitation.

### Flow cytometry detection

WT or APP/PS1 Tg mice were killed using tribromoethanol and perfused using PBS, and the hippocampus and cerebral cortex were removed. The obtained tissues were washed twice in DMEM serum-free medium and then quickly cut into 1 mm^3^ pieces using a scalpel. The cells were incubated at 37 °C in 5 ml DMEM containing 2.5 mg trypsin and 5 mg collagenase for 20 min. The digestive reaction was stopped by adding DMEM containing 10% serum, and the cell suspension was strained through a 40 μm cell sieve. The filtrate was centrifuged and resuspended in 6 ml HSSS and carefully placed on top of the Optiprep gradient solution. The tubes containing the cells and gradient solution were centrifuged in a Thermo Fisher centrifuge for 15 min at 1900 rpm. The lipid and debris layers were carefully aspirated, and the glial layer at the bottom was resuspended in 2.5 ml HBSS solution. After washing with HBSS, cells were fixed in 4% paraformaldehyde for 1 h and then incubated in 1% Triton X-100 for 1 h. Finally, OPTN and Iba1 antibodies were added and incubated at 4 °C for 4 h after blocking with goat serum for 1 h. After washing with PBS, the fluorescent secondary antibody was added and incubated at room temperature for 1 h. The samples were analyzed using a FACSCalibur (Becton–Dickinson) flow cytometer. For all samples, 5000 events per sample were read, and Flow Jo was used to complete the data analysis.

### Measurement of the IL-1β concentration

The IL-1β levels in the media of both the control cells and the Aβo-treated cells were determined using the IL-1β enzyme immunoassay kits obtained from R&D Systems (Minneapolis, MN, USA), following the manufacturer’s instructions. The results were expressed as ng IL-1β per ml medium.

### Quantitative real-time PCR (qRT-PCR)

Real-time PCR assays were performed on the MiniOpticon Real-Time PCR detection system (Bio-Rad) using total RNA and the GoTaq One-step Real-Time PCR kit with SYBR green (Promega, Madison, WI, USA) [52]. The gene expression levels were normalized to those of glyceraldehyde-3-phosphate dehydrogenase (GAPDH). GAPDH (NM_001289726.1) F-AACTTTGGCATTGTGGAAGG, R-ACACATTGGGGGTAGGAACA; AIM2 (NM_001013779) F-TGGAGGTCACCAGTTCCTCA, R-TTCCTCTGTTATCTTCTGGACTTT; ASC (NM_023258) F-GTCGTATGGCTTGGAGCTCA, R-CCACAGCTCCAGACTCTTCT; OPTN (NM_001356487) F-GGAGGCAGTAGACAGTCCCT, R-CACTTGGGGCAGGAGTGAAT; RIPK1 (NM_001359997) F-GCCAGTAGCAGATGACCTCA, R-GCTTGGTGTCTGGAAGTCGA. The ratio was calculated using the following equation:

$${\text{Ratio}} = \frac{{2^{{\Delta {\text{Ct}}({\text{Gene}}_{{{\text{Control}}}} - {\text{Gene}}_{{{\text{Experiment}}}} )}} }}{{2^{{\Delta {\text{Ct}}({\text{GAPDH}}_{{{\text{Control}}}} - {\text{GAPDH}}_{{{\text{Experiment}}}} )}} }}.$$

The untreated sample was always set to 1, and the value of the treatment group was obtained from the previous equation.

### Western blotting analysis

Tissues or cells were lysed in RIPA buffer (25 mM Tris–HCl [pH 7.6], 150 mM NaCl, 1% Nonidet P-40, 1% sodium deoxycholate, and 0.1% SDS), containing a protease inhibitor cocktail (Thermo Fisher Scientific, Waltham, MA, USA). The protein content of the cell lysates was determined using a bicinchoninic acid protein assay reagent (Thermo Fisher Scientific, Waltham, MA, USA). The total cell lysates (15 μg) were subjected to SDS-PAGE, transferred to a membrane, and incubated with a panel of specific antibodies. Each membrane was probed with only one antibody, and β-actin was used as a loading control. All western blotting experiments were performed at least in triplicate, with a different cell preparation each time.

### Immunohistochemistry (IHC)

Mouse brains were collected from 3-month-old APP/PS1-Control-AAV or APP/PS1-OPTN-AAV Tg mice and immobilized with 4% paraformaldehyde. Serial sections (thickness: 10 μm) were cut using a cryostat (CM1850; Leica, Wetzlar, Germany). The slides were rehydrated in a graded series of ethanol and submerged in 3% hydrogen peroxide, to eliminate the activity of endogenous peroxidase. The levels of Iba1 and GFAP were determined using an immunohistochemical staining kit, according to the instructions provided by the manufacturer (Invitrogen, Carlsbad, CA, USA) [53].

### Preparation of lentiviral particles

Lentiviral vectors encoding the mouse OPTN, HA-OPTN, and Flag-RIPK1 genes, as well as a control lentiviral vector were provided by Keygen Biotech. Co. (Nanjing, China). The lentiviral vectors were purified and co-transfected with packaging vectors (psPAX_2_ and pMD_2_.G) into HEK293T cells. After 48 h and 72 h, the lentiviral particles in the supernatant were concentrated through ultracentrifugation and resuspended in PBS (−). For knocking down the expression of OPTN, the lentiviral particles that contained sh-OPTN or control shRNA were adjusted to 10^6^–10^7^ titers prior to infecting BV2 cells.

### Purification of adeno-associated virus (AAV)

Recombinant AAV-OPTN was generated via triple transfection of HEK293T cells with pAOV-OPTN, pAAV-RC9, and pHelper vectors using Lipofectamine 2000 Thermo Fisher Scientific (Waltham, MA, USA), Waltham, MA, USA). Viral particles were harvested from the media 72 h after transfection. Cell pellets were resuspended in 10 mM Tris with 2 mM magnesium dichloride, pH 8, freeze-thawed three times, and treated with 100 U/ml Benzonase at 37 °C for 1 h. After centrifugation at 13,000 *g* for 10 min, the supernatants were collected. The combined media and supernatants were concentrated through precipitation with 10% polyethylene glycol 8000 (Sigma-Aldrich, St. Louis, MO, USA) and 500 mM NaCl. After centrifugation at 15,000 *g* for 30 min, the precipitated virus was suspended in 10 mM Tris with 2 mM magnesium dichloride. The virus particles were purified using gradient (15%, 25%, 40%, and 60%) iodixanol (Sigma-Aldrich, St. Louis, MO, USA). The viral titers were determined through qRT-PCR. The results were expressed as DNA resistance particles/ml.

### Intracerebroventricular (i.c.v) injection of AAV-OPTN

Particles of AAV-OPTN viruses were injected (i.c.v) to APP/PS1 mice. In brief, stereotaxic injections were performed at the following coordinates from the bregma: mediolateral, 2.10 mm; anteroposterior, 2.00 mm; and dorsoventral, 2.28 mm. Following injection, each mouse was allowed to spontaneously recover on a heated pad. The reliability of the injection sites was validated by injecting trypan blue dye (Invitrogen, Carlsbad, CA, USA) in separate cohorts of mice and observing the staining in the cerebral ventricles. At 25 days post-injection, the mice were euthanized while under anesthesia and perfused with PBS (−) [54].

### Transfection

Plasmids for HA-tagged OPTN and their fragment, Flag-tagged RIPK1 and their fragment were cloned into the plvx-IRES-zsgreen vector for transient expression in HEK293T cells using Lipofectamine 2000 (Invitrogen, Carlsbad, CA, USA). In the control experiments, plvx-IRES-zsgreen plasmids were transfected to BV2 cells via similar methods. The transfected cells were allowed to recover for ≥ 12 h in growth medium and subsequently incubated overnight in serum-free medium prior to treatment with Aβo before extraction.

### Infection with lentiviral particles

BV2 cells were seeded in 24-well plates at a density of 2 × 10^5^ cells/well. Lentiviral particles and 8 μg/ml polybrene (Sigma-Aldrich, St. Louis, MO, USA) were added to the culture and centrifuged for 90 min at 1.5 × 10^3^ rpm. The supernatant was removed immediately after infection and replaced with basal medium (Invitrogen, Carlsbad, CA, USA) containing 10% fetal bovine serum and 50% conditional medium. The efficiency of the infection was determined via qRT-PCR and western blotting after 72 h.

### Animal management

All animals were managed according to the Care and Use of Medical Laboratory Animals (Ministry of Health, Beijing, China) and all experimental protocols were approved by the Laboratory Ethics Committee of Northeastern University of China.

### Statistical analysis

All data are presented as the mean ± standard error of at least three independent experiments. The statistical significance of the differences between the means was determined using Student’s *t* test or one-way analysis of variance, as appropriate. In cases of significantly different means, multiple pairwise comparisons were performed using Tukey’s post hoc test.

## Results

### Expression of OPTN is downregulated in the brains of AD patients and APP/PS1 Tg mice

**Fig. 1:**
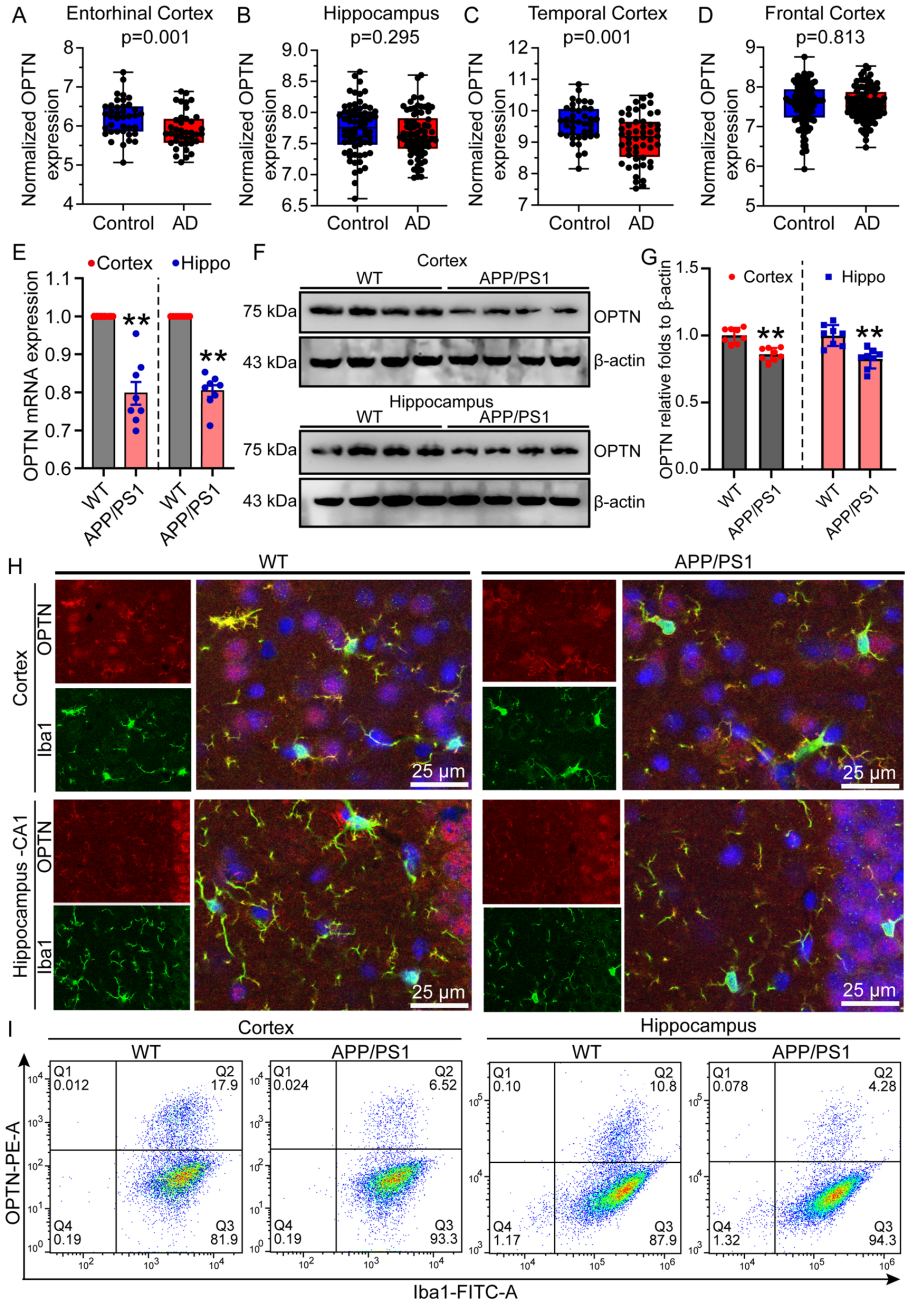
OPTN expression is downregulated in AD patients and APP/PS1 transgenic mice. **A**–**D** Transcriptome data of the entorhinal cortex, hippocampus, frontal cortex, and temporal cortex in AD patients were analyzed after normalization. **E**–**H** Nine-month-old APP/PS1 transgenic mice were anesthetized and euthanized to obtain the cerebral cortex and hippocampus. **E** Expression of OPTN in the cerebral cortex and hippocampus of APP/PS1 transgenic mice was detected by qRT-PCR using GAPDH as an internal control. **F** The protein level of OPTN in the cerebral cortex and hippocampus of APP/PS1 transgenic mice was assessed by western blotting using β-actin as an internal control. **G** ImageJ software was used to semiquantitatively analyze the fold change of OPTN relative to β-actin. **H** WT or APP/PS1 Tg mice were double-stained for Iba1 (green) and OPTN (red). **I** Expression of OPTN in microglia in the cortex and hippocampus of APP/PS1 transgenic mice at 9 months of age was detected by flow cytometry. The data represent the means ± S.E. of independent experiments. APP/PS1 transgenic mice were compared with WT mice *P < 0.05, **P < 0.01

With increasing age, cells gradually become senescent, resulting in the disruption of various cell functions, such as overloading the degradation ability of the proteasome, leading to activation of the autophagy system to remove accumulated misfolded proteins, intracellular aggregates and irreparably damaged organelles [55]. In addition, many proteins that are mutated in neurodegenerative diseases are related to autophagy or lysosomal function [56, 57]. Given these observations, we searched the GEO database to integrate transcriptome sequencing data of AD patients. All of the collected data were divided into four groups: the entorhinal cortex (GSE26927, GSE48350, GSE5281, GSE26972), frontal cortex (GSE12685, GSE15222, GSE33000, GSE36980, GSE48350, GSE5281), hippocampus (GSE28146, GSE29378, GSE36980, GSE48350, GSE5281), and temporal cortex (GSE15222, GSE36980, GSE5281, GSE37263). As an autophagy receptor, the OPTN gene was identified to be significantly decreased in AD patients compared to healthy controls (Fig. 1A–D). To further validate these results, we measured the expression of OPTN in 9-month-old APP/PS1 Tg mice. The results demonstrated that the mRNA and protein expression of OPTN was downregulated in the cerebral cortex and hippocampus of 9-month-old APP/PS1 Tg mice compared to that of WT mice (Fig. 1E–G), which is consistent with the transcriptomic sequencing data from AD patients (Fig. 1A–D).

Because OPTN expression is downregulated in the brains of AD patients and APP/PS1 Tg mice, we further determined its origin. For this purpose, a double-immunofluorescence labeling technique was used to determine the localization of OPTN in the brains of 9-month-old APP/PS1 Tg mice. By immunostaining the brains with OPTN together with either NeuN, Iba1 or GFAP, we found that OPTN colocalized with Iba1 (Fig. 1H), and its expression in microglial cells was downregulated, as shown by flow cytometry (Fig. 1I). This result suggests that OPTN is primarily expressed in microglial cells but not neurons or astrocytes in the brains of 9-month-old APP/PS1 Tg mice.

### OPTN is critical for mitophagy

**Fig. 2:**
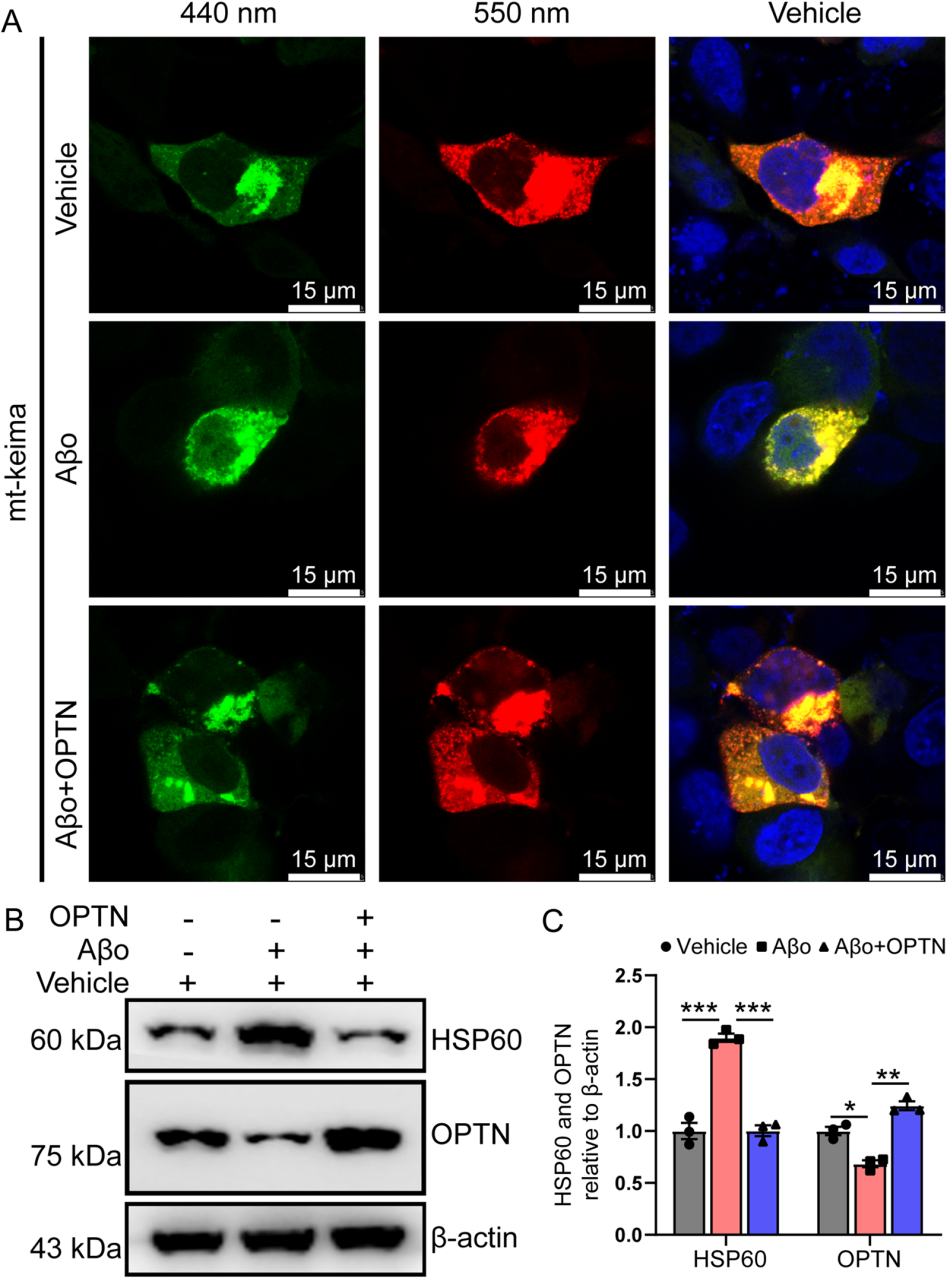
OPTN alleviates Aβo-induced dysfunction of mitochondrial autophagy. **A**, **B** BV2 cells were treated with Aβo in the absence or presence of transfection with plvx-IRES-OPTN. **A** mKeima fluorescence was evoked using two excitation filters (438 ± 12 nm and 550 ± 15 nm) and a 610LP emission filter. **B** Expression levels of HSP60 and OPTN were detected by western blotting. β-actin served as an internal control. The data represent the means ± S.E. of independent experiments. BV2 cells treated with vehicle group or Aβo and overexpressing OPTN were compared with group Aβo alone *P < 0.05, **P < 0.01, ***P < 0.001

As an essential receptor for mitophagy [58], we initially investigated the roles of OPTN in autophagy. By overexpressing the mitochondrial keima protein in BV-2 cells, we found that Aβo treatment markedly blocked the fusion between mitochondria and lysosomes, as evidenced by attenuation of the red fluorescent signals at 543 nm, which is activated by the acidic environment of the lysosomal cavity (Fig. 2A). When we overexpressed OPTN in Aβo-treated BV-2 cells, the red fluorescence was restored, indicating the recovery of autophagic function (Fig. 2A). To further validate these observations, we measured the expression of heat shock protein 60 (HSP60). Using western blot, we revealed that OPTN overexpression blocked the effects of Aβo on inducing the protein expression of HSP60 in treated BV2 cells (Fig. 2B). Based on these observations, OPTN was identified to be essential for mitophagy.

### AIM2 inflammasomes are activated during the progression of AD

**Fig. 3:**
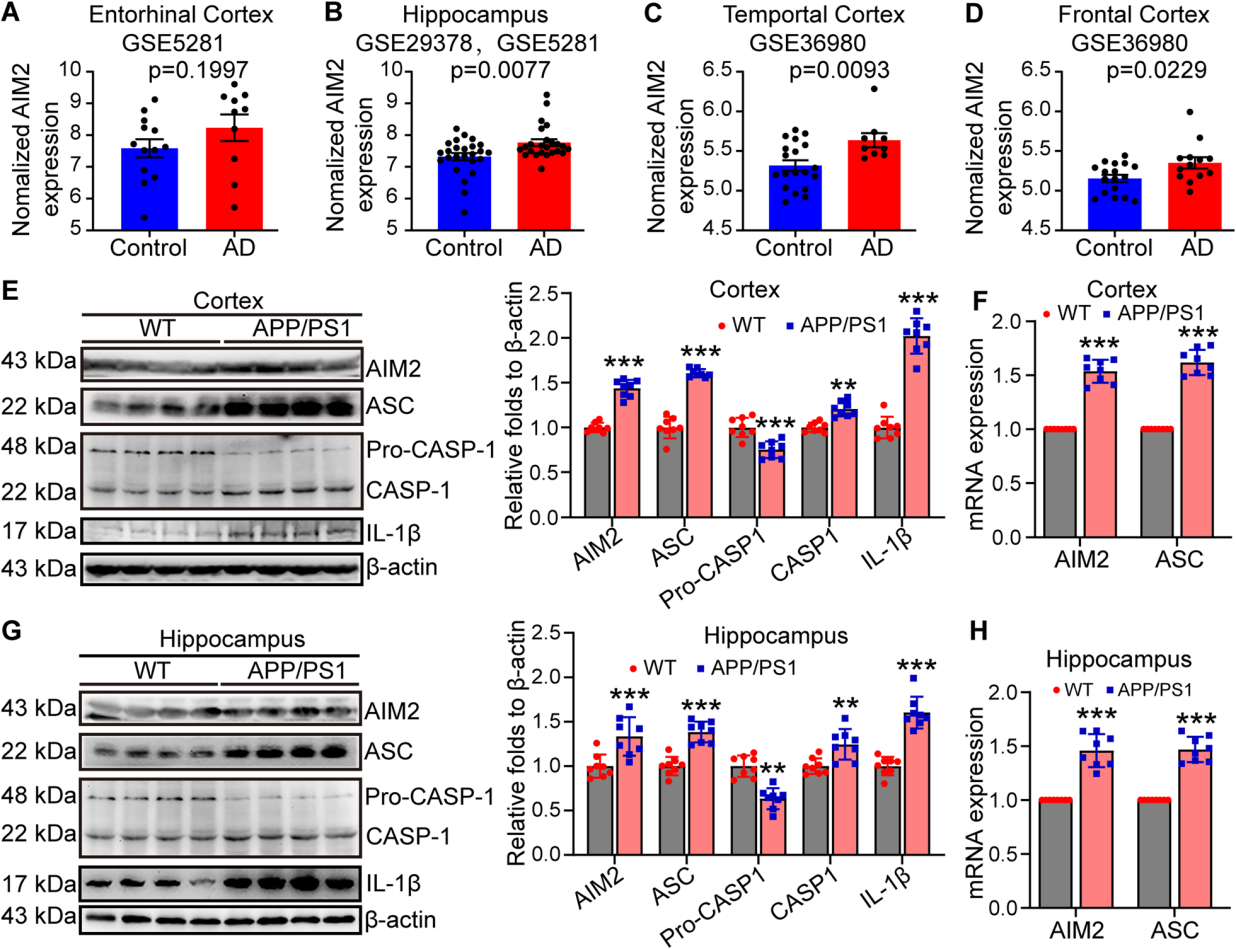
The AIM2 inflammasome is activated in AD patients and APP/PS1 transgenic mice. **A**–**D** Brain transcriptome data from patients with AD and controls were collected from the GEO database and normalized for analysis. **E**–**H** APP/PS1 transgenic mice at the age of 9 months were anesthetized and euthanized to obtain the cerebral cortex and hippocampus. **E** Detection of the expression levels of AIM2, ASC, pro-caspase-1, caspase-1 and IL-1β in the cerebral cortex by western blotting. β-actin served as the internal control. In the right panel, ImageJ software was used to semiquantitatively analyze the western blotting results. **F** qRT-PCR was used to detect the mRNA expression of AIM2 and ASC in the cerebral cortex. GAPDH served as internal control. **G** The expression of AIM2, ASC, pro-caspase-1, caspase-1 and IL-1β in the hippocampus was detected by western blotting. β-actin served as the internal control. **H** The mRNA expression of AIM2 and ASC in the hippocampus was detected by qRT-PCR. GAPDH was used as the internal control. The data present means ± S.E. of independent experiment. APP/PS1 transgenic mice were compared with WT mice *P < 0.05, **P < 0.01, ***P < 0.001

By disrupting mitochondrial autophagy, impaired mitochondria release reactive oxygen species (ROS), free radicals and mitochondrial DNA (mtDNA) into the cytoplasm due to a lack of mitochondrial clearing mechanisms, potentially contributing to inflammation [59]. Indeed, AIM2 has been reported to bind to free cytoplasmic DNA through its HIN200 domain, resulting in the oligomerization of ASC and leading to the formation of caspase-1-dependent inflammatory bodies and the maturation and secretion of proinflammatory cytokines, such as IL-1β and IL-18 [60]. For these reasons, we assessed the activity of AIM2 inflammasomes during the course of AD development and progression. By analyzing the GEO database, expression of AIM2 and ASC was found to be upregulated in AD patients compared to healthy controls (Fig. 3A–D). To confirm the results in the database, we further determined the mRNA and protein expression of AIM2 inflammasome components, including AIM2, ASC, caspase-1 and IL-1β, in the cerebral cortex and hippocampus of 9-month-old APP/PS1 Tg mice. Using qRT-PCR and western blots, the results demonstrated that mRNA and protein expression of AIM2 and ASC was upregulated in the cerebral cortex and hippocampus of APP/PS1 Tg mice compared to WT controls (Fig. 3E–H). The cleaved active form of caspase-1 is produced from pro-caspase-1 in APP/PS1 Tg mice (Fig. 3E, G). Moreover, the protein abundance of mature IL-1β was enhanced in APP/PS1 Tg mice (Fig. 3E, G). These findings indicate that AIM2 inflammasomes are activated in the AD brains.

### Aβo activates AIM2 inflammasomes

**Fig. 4:**
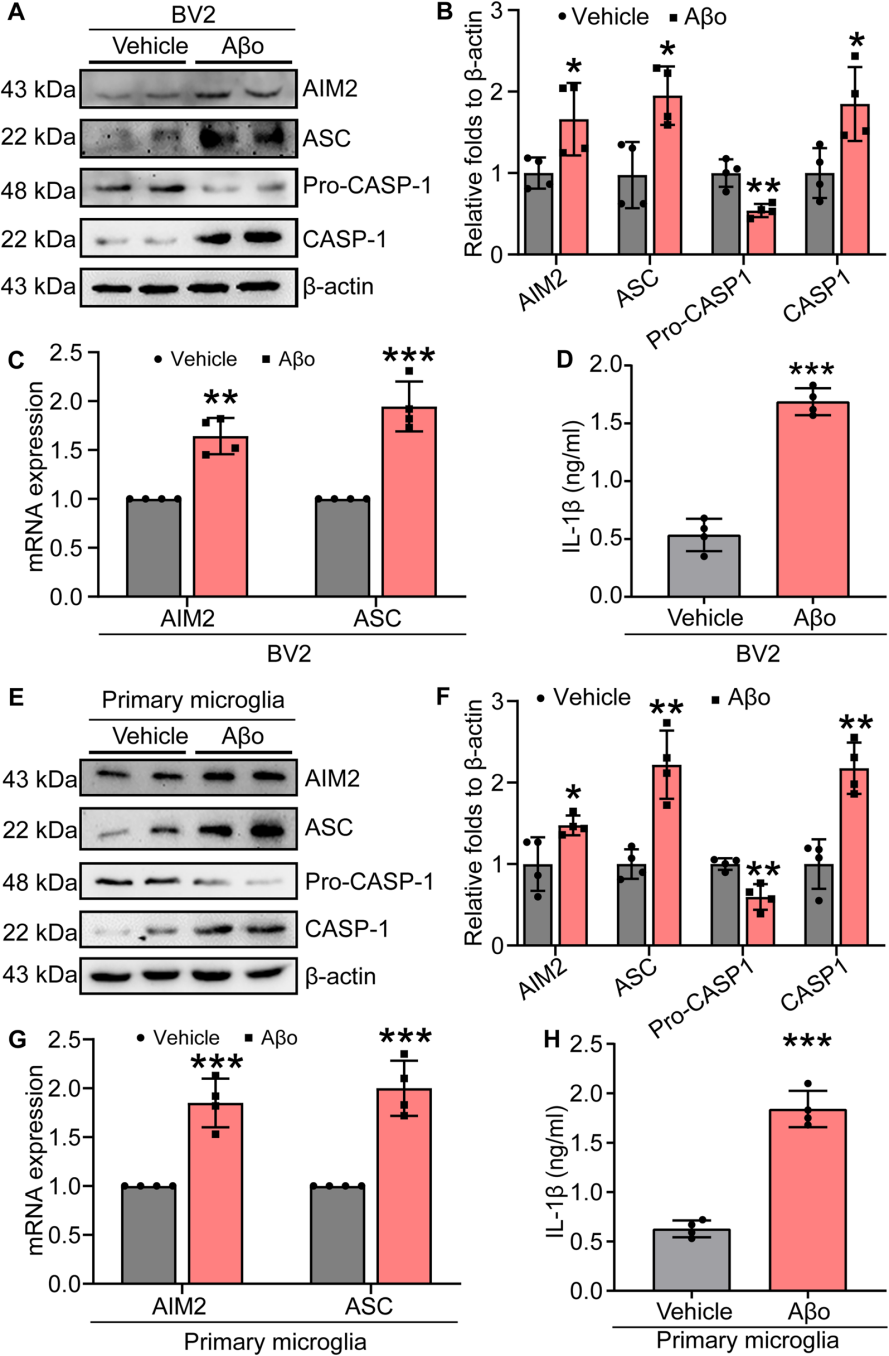
Aβo activates the AIM2 inflammasome. **A**–**D** BV2 cells were treated with Aβo for 12 h. **A** Expression levels of AIM2, ASC, pro-caspase-1 and caspase-1 were detected by western blotting. β-actin served as an internal control. **B** ImageJ software was used for semiquantitative analysis of western blots. **C** qRT-PCR was used to detect the mRNA expression of AIM2 and ASC with GAPDH as an internal control. **D** Secretion of IL-1β was evaluated by ELISA. **E**–**H** Primary microglia were treated with Aβo for 12 h. **E** Protein levels of AIM2, ASC, pro-caspase-1, and caspase-1 were detected by western blotting with β-actin as the internal control. **F** ImageJ software was used to semiquantitatively analyze the fold change in AIM2, ASC, pro-caspase-1 and caspase-1 relative to β-actin. **G** mRNA expression of AIM2 and ASC was detected by qRT-PCR with GAPDH as an internal control. **H** Secretion of IL-1β was detected by ELISA. The data present means ± S.M. of independent experiment. Aβo treatment were compared with vehicle treatment *P < 0.05, **P < 0.01, ***P < 0.001

Since the AIM2 inflammasome was activated in the brains of AD patients and APP/PS1 Tg mice, we logically next explored the effects of Aβ on the activation of inflammasomes. In BV2 cells, Aβo treatment clearly induced mRNA and protein expression of both AIM2 and ASC (Fig. 4A, C). Similarly, the active form of caspase-1 and mature IL-1β were produced in Aβo-treated BV-2 cells (Fig. 4A, B, D). To further validate these observations, we performed similar experiments in primary cultured microglial cells treated with Aβo, and similar results were obtained (Fig. 4E–H). These results clearly indicate that Aβo activates AIM2 inflammasomes during the course of AD development and progression.

### OPTN blocks the effects of Aβo on activating AIM2 inflammasomes

**Fig. 5:**
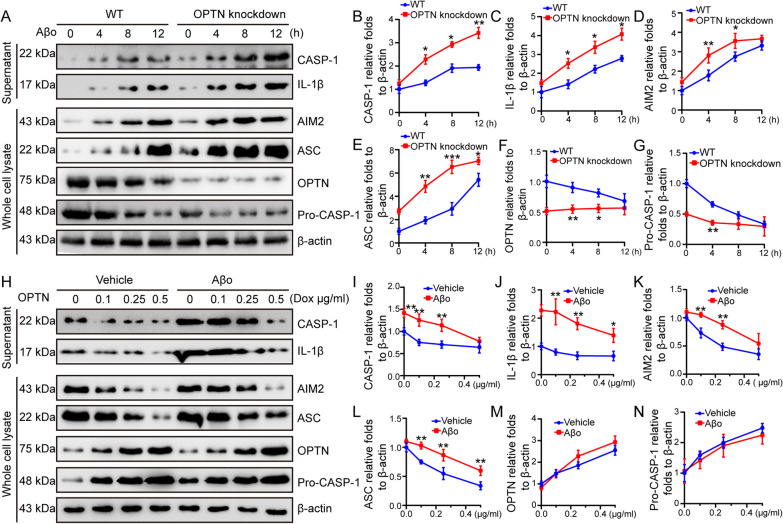
OPTN alleviates the Aβo-induced AIM2 inflammasome. **A**–**G** OPTN was silenced in BV2 cells that were then treated with Aβo for 4, 8, and 12 h. **A** Western blotting was used to detect the protein expression of AIM2, ASC, OPTN and pro-caspase-1 in whole-cell lysates. Meanwhile, extracellular secretion of caspase-1 and IL-1β was assessed in the conditioned medium. **B**–**G** ImageJ software was used to semiquantitatively analyze the fold changes in caspase-1, IL-1β, AIM2, ASC, OPTN and pro-caspase-1 relative to β-actin. The data are presented as the mean ± S.M. of independent experiment. WT BV2 cells compared to OPTN knockdown, *P < 0.05, **P < 0.01, ***P < 0.001. **H**–**N** BV2 cells gradually increased their expression of OPTN in the absence or presence of Aβo for 12 h. **H** Western blotting was used to detect the protein expression of AIM2, ASC, OPTN and pro-caspase-1 in whole-cell lysates. Meanwhile, extracellular secretion of caspase-1 and IL-1β was detected in the cell culture medium. **I**–**N** ImageJ software was used to semiquantitatively analyze the fold change in caspase-1, IL-1β, AIM2, ASC, OPTN and pro-caspase-1 relative to β-actin. The data are presented as the means ± S.M. of independent experiment. The data present mean ± S.M. of independent experiment. Aβo treatment BV2 cells compared with vehicle treatment BV2 cells, *P < 0.05, **P < 0.01, ***P < 0.001

Given the potential roles of OPTN in neuroinflammation [61], we further investigated its roles in Aβo-activated AIM2 inflammasomes. By knocking down the expression of OPTN in BV2 cells, Aβo exhibited enhanced ability to increase the active cleavage product of caspase-1 and mature IL-1β in supernatants (Fig. 5A–C). In whole-cell lysates, Aβo induced early activation of AIM2 and ASC and decreased the protein abundance of pro-caspase-1 in OPTN knockdown BV2 cells (Fig. 5A, D–G). These results revealed that OPTN deficiency facilitated the activation of AIM2 inflammasomes in Aβo-stimulated microglial cells.

Reciprocally, we ectopically expressed OPTN in BV2 cells. In response to increasing OPTN protein levels, the active form of caspase-1 and the production of mature IL-1β were decreased in OPTN-overexpressing BV2 cells (Fig. 5H–J). In whole-cell lysates, the protein abundance of AIM2 and ASC was downregulated, and pro-caspase-1 accumulated due to the lack of cleavage into the active form of caspase-1 in OPTN-overexpressing cells (Fig. 5H, K–H). Although Aβo can activate AIM2 inflammasomes, it was unable to diminish the effects of OPTN overexpression on deactivating AIM2 inflammasomes (Fig. 5H–L). Based on these observations, OPTN alleviates the effects of Aβo on activating AIM2 inflammasomes in microglial cells.

### As a downstream target of TLRs, RIPK1 expression is elevated in the brains of AD patients

**Fig. 6:**
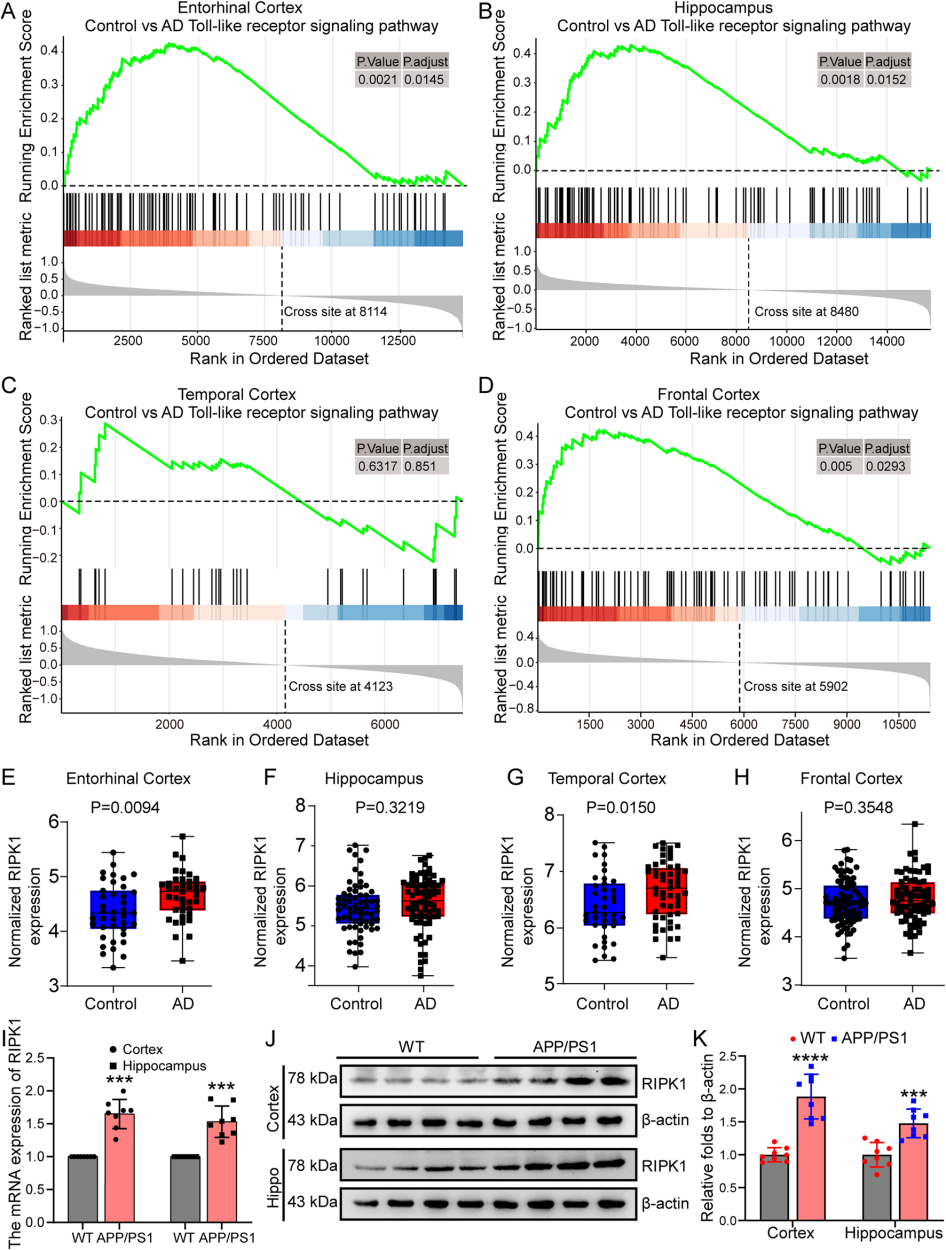
The Toll-like receptor pathway is upregulated in AD patients, and RIPK1 expression is increased in APP/PS1 transgenic mice. **A**–**D** Transcriptome sequencing data of the entorhinal cortex, hippocampal cortex, temporal cortex and frontal cortex tissues from AD patients were collected from the GEO database, and GSEA was performed after normalization. **E**–**H** The differences in RIPK1 expression in the transcriptome of the entorhinal cortex, hippocampus, frontal cortex and temporal cortex were normalized in AD patients. **I**–**K** Nine-month-old APP/PS1 Tg mice were anesthetized and euthanized to obtain the cerebral cortex and hippocampus. **I** mRNA expression of RIPK1 in the cerebral cortex and hippocampus was detected by qPCR using GAPDH as the internal control. **J** Protein levels of RIPK1 in the cerebral cortex and hippocampus were elucidated by western blotting with β-actin as the internal control. **k** ImageJ software was used to semiquantitatively analyze the fold change in RIPK1 relative to β-actin. The data present means ± S.M. of independent experiment. The data present mean ± S.M. of independent experiment. APP/PS1 Tg mice compared with WT mice, ***P < 0.001

As discussed above, TLRs mediate the effects of ligands or endogenous stimulators, such as Myd88 and TRIF, on activating NF-κB or AP-1, which results in the activation of inflammasomes [41]. Specifically, TLR2, 4 and 6 mediate the effects of Aβ_1–42_ fibrils on promoting the maturation and secretion of tumor necrosis factor α (TNF-α) and IL-1β in microglial cells [23, 62]. Moreover, LPS, the natural ligand of TLR4, has been proposed to activate inflammasomes via the Toll-like receptor adaptor molecule 1 (TICAM1/TRIF)-, receptor interacting serine/threonine kinase 1 (RIPK1)-, FAS-associated death domain protein (FADD)- and caspase-8-axes [43]. Based on these clues, we next explored the activity of TLRs in AD. GSEA of transcriptome sequencing data from AD patients in the GEO database demonstrated that TLR signaling pathways were markedly upregulated in the entorhinal cortex, frontal cortex and hippocampus (Fig. 6A–D). As a downstream target of TLRs, RIPK1 in this pathway plays critical roles in driving inflammation, apoptosis and necrosis [49, 63]. In addition, RIPK1 activation has been reported to be associated with neurodegenerative diseases via inflammation-activating mechanisms [49]. Therefore, it is necessary to determine the activity of RIPK1 during the course of AD development and progression. As expected, we found that expression of RIPK1 was significantly upregulated in the entorhinal and temporal cortex of AD patients compared to healthy controls (Fig. 6E, G). Even though it was not statistically significant, the average expression of RIPK1 was higher in the hippocampus and frontal cortex in AD patients than in healthy subjects (Fig. 6F, H). To further validate these observations, we determined the expression of RIPK1 in the brains of APP/PS1 Tg mice. The results demonstrated that mRNA and protein expression of RIPK1 was elevated in the cerebral cortex and hippocampus of APP/PS1 Tg mice compared to WT mice (Fig. 6I–K). These observations indicate that the signaling cascades of TLRs and RIPK1 are activated during the course of AD development and progression.

### OPTN deactivates the neuroinflammatory pathways of RIPK1

**Fig. 7:**
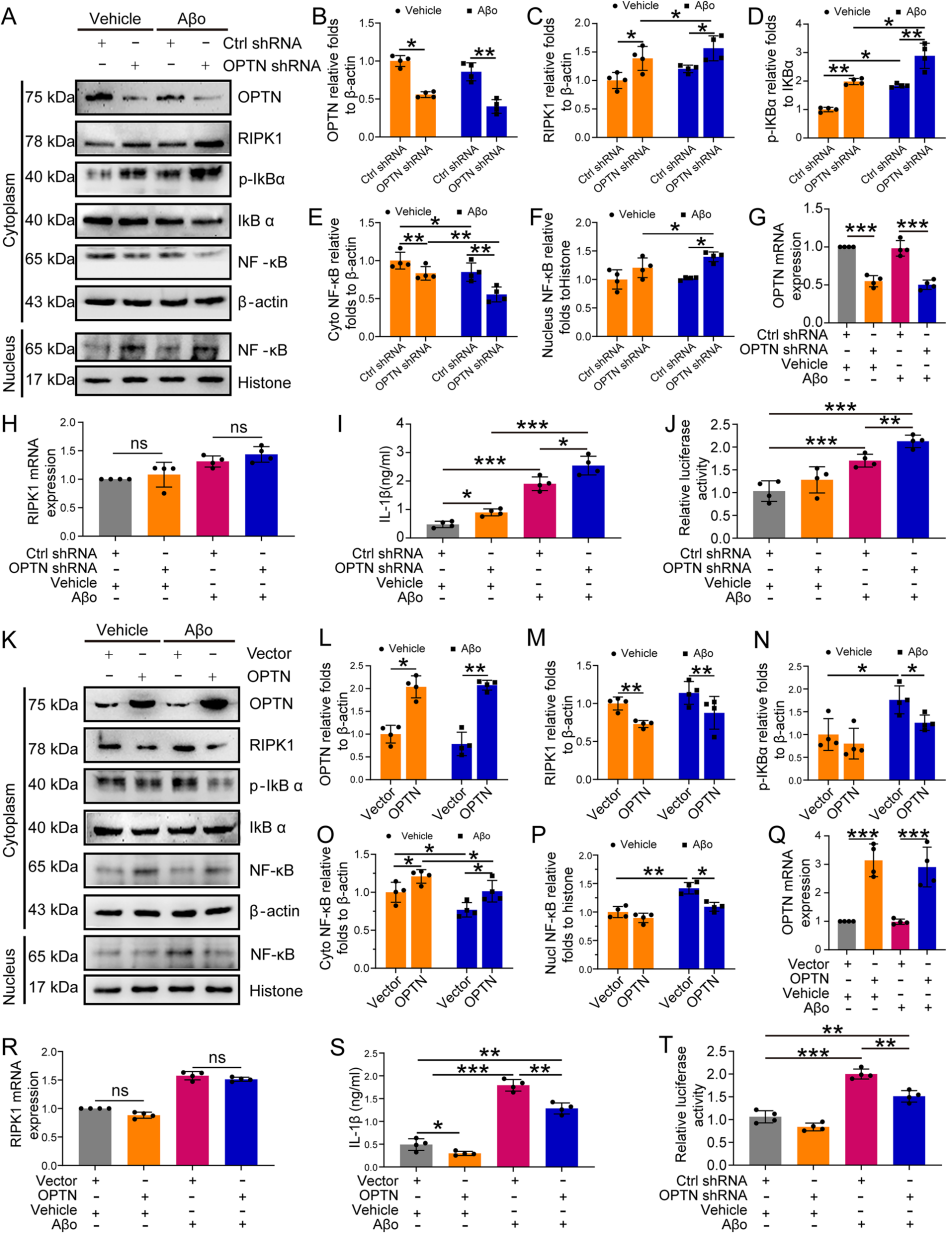
OPTN negatively regulates RIPK1 inflammatory signaling pathways. **A** BV2 cells with OPTN silenced were treated with Aβo for 12 h. Then, OPTN, RIPK1, p-IκBα, and IκBα in the cytoplasm and NF-κB in the cytoplasm or nucleus were detected by western blot with β-actin as an internal control. **B**–**G** ImageJ software was used to semiquantitatively analyze the optical density of western blots. **H**–**K** OPTN-silenced BV2 cells were treated with Aβo for 12 h. **H** OPTN mRNA expression was detected by qRT-PCR using GAPDH as an internal control. **I** RIPK1 RNA expression was detected by qRT-PCR using GAPDH as an internal control. **J** Extracellular secretion of IL-1β was assessed by ELISA. **K** The binding activity of NF-κB was evaluated by dual-luciferase assay. The data are presented as the means ± S.M. of independent experiment. OPTN-silenced BV2 cells compared to control BV2 cells or Aβo-treated BV2 cells compared to vehicle BV2 cells, *P < 0.05, **P < 0.01, ***P < 0.001. **L**–**V** BV2 cells with ectopic overexpression of OPTN in the absence or presence of Aβo treatment for 12 h. **L** Protein levels of OPTN, RIPK1, p-IκBα, and IκBα in the cytoplasm and NF-κB in the cytoplasm or nucleus were detected by western blot using β-actin as an internal control. **N**–**R** ImageJ software was used to semiquantitatively analyze the western blot results. **S** mRNA expression of OPTN was detected by qRT-PCR using GAPDH as an internal control. **T** mRNA expression of RIPK1 was detected by qRT-PCR using GAPDH as an internal control. **U** Extracellular secretion of IL-1β was assessed by ELISA. **V** The binding activity of NF-κB was evaluated using a dual-luciferase assay. The data present means ± S.M. of independent experiment. OPTN overexpressed BV2 cells compared with control BV2 cells or Aβo-treated BV2 cells compared with vehicle BV2 cells, *P < 0.05, **P < 0.01, ***P < 0.001

Since OPTN has been shown to depress the activities of AIM2 inflammasomes, we further elucidated its roles in regulating RIPK1-mediated inflammatory pathways. As a result, knocking down the expression of OPTN induced the accumulation of RIPK1 protein in the cytoplasm of BV2 cells (Fig. 7A–C). Overloading RIPK1 in the cytoplasm triggers the translocation from the cytoplasm to the nucleus by enhancing the phosphorylation of IκBα in BV2 cells (Fig. 7A, D–G). Interestingly, the mRNA expression of RIPK1 was not changed by knocking down the expression of OPTN in BV2 cells (Fig. 7H–I). Based on these observations, we further determined the roles of OPTN in the production of IL-1β in BV2 cells. Using ELISA, we determined that shRNA-mediated knockdown of OPTN expression elevated the production of IL-1β in BV2 cells (Fig. 7J). For this reason, knocking down the expression of OPTN promoted the transcriptional activity of NF-κB in BV2 cells (Fig. 7K). In OPTN knockdown cells, we further treated BV2 cells with Aβo for 12 h. Compared with to controls, Aβo treatment markedly induced protein accumulation of RIPK1 by slightly depressing the protein levels of OPTN in BV-2 cells (Fig. 7A–C). As a consequence, NF-κB translocates from the cytoplasm to the nucleus by enhancing the phosphorylation of IκBα, which results in activating the transcriptional activity of NF-κB, leading to the synthesis of IL-1β in BV-2 cells (Fig. 7A, D–G, J, K). More interestingly, the mRNA expression of RIPK1 was elevated in response to treatment with Aβo in BV2 cells (Fig. 7I).

To further validate the above observations, we overexpressed OPTN in BV2 cells. Ectopic overexpression of OPTN resulted in markedly decreased protein levels of RIPK1 without affecting the mRNA levels of RIPK1 in BV2 cells (Fig. 7L–N, S, T), leading to reduce translocation of NF-κB from the cytoplasm to the nucleus by dephosphorylating IκBα in BV2 cells (Fig. 7L, O–R). By depressing the transcriptional activity of NF-κB, the production of IL-1β was decreased in OPTN-overexpressing BV2 cells (Fig. 7U, V). In OPTN-overexpressing cells, we further treated BV2 cells with Aβo for 12 h. Treatment with Aβo blocked the effects of Aβo on inducing the protein accumulation of RIPK1 in BV2 cells (Fig. 7L–N). Similarly, phosphorylation of IκBα was also prevented by OPTN overexpression, which impaired NF-κB translocation from the cytoplasm to the nucleus in Aβo-treated BV2 cells (Fig. 7L, O–R). Consistent with these observations, OPTN overexpression partially suppressed the ability of Aβo to induce the synthesis of IL-1β and the transcriptional activity of NF-κB in BV2 cells (Fig. 7U, V). Notably, OPTN overexpression did not block the effects of Aβo on inducing the synthesis of IL-1β or the transcriptional activity of NF-κB in BV2 cells (Fig. 7U, V). Based on these observations, the NF-κB p65 subunit might not be a unique factor for the transcriptional activity of NF-κB or the secretion of IL-1β in BV2 cells.

### OPTN promotes ubiquitin protease hydrolysis of RIPK1 through ubiquitination

**Fig. 8:**
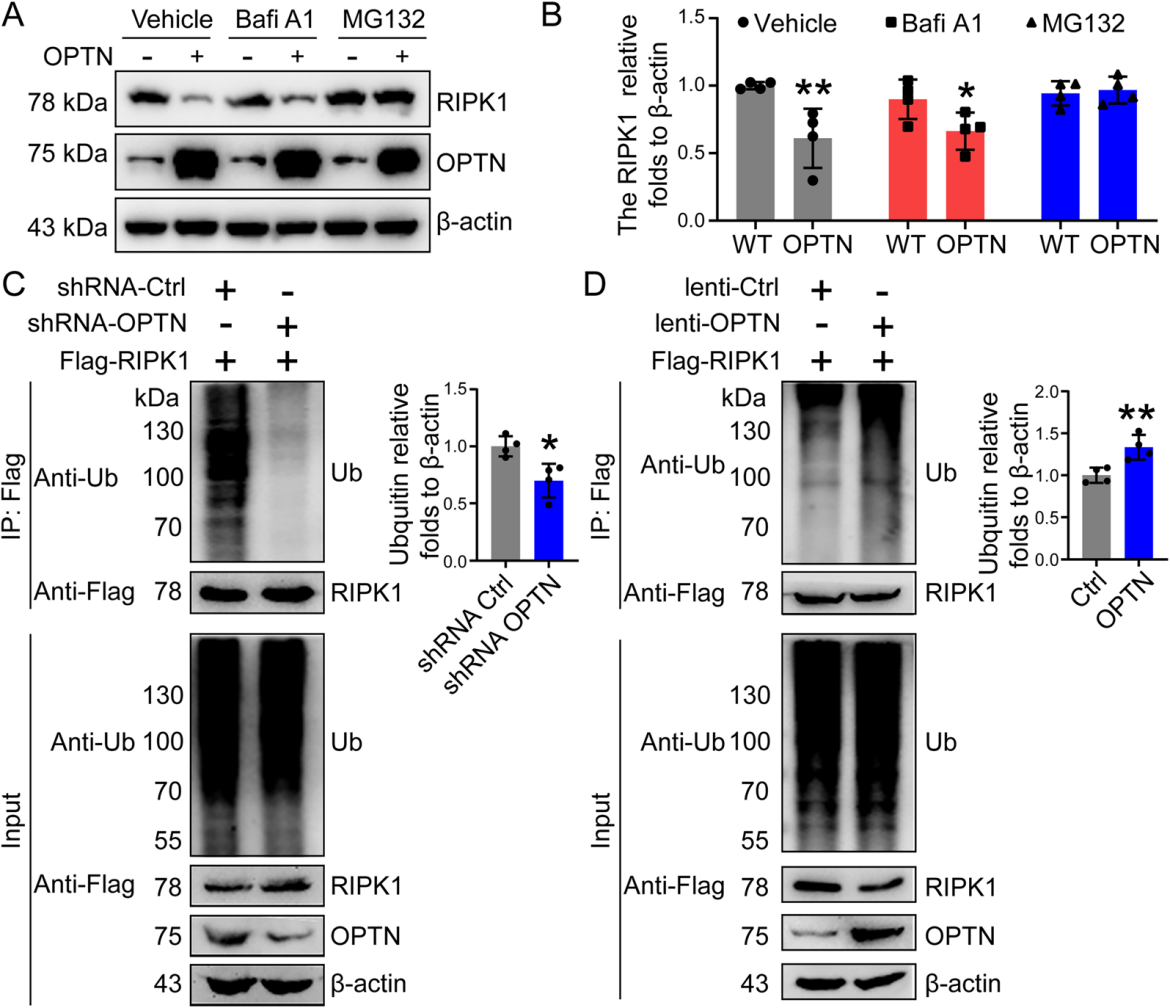
OPTN degrades RIPK1 through the ubiquitin proteasome pathway. **A** BV2 cells overexpressing OPTN were treated with bafilomycin A1 (200 nM) or MG132 (10 μM) for 6 h. The expression of RIPK1 and OPTN was detected by western blotting using β-actin as an internal control. **B** ImageJ software was used to semiquantitatively analyze the western blot results. **C** Flag-RIPk1 was ectopically expressed in OPTN-silenced BV2 cells, which were then immunoprecipitated using an anti-Flag antibody derived from mice. The precipitated protein was further detected using a ubiquitin antibody derived from rabbits. Flag antibody was used to detect RIPK1, and OPTN was used to detect the OPTN protein levels in the total protein. In the right panel, ImageJ software was used to semiquantitatively analyze ubiquitin levels. **D** OPTN was ectopically overexpressed in Flag-RIPk1-overexpressing BV2 cells, which were then immunoprecipitated using an anti-Flag antibody derived from mice. The precipitated protein was detected using rabbit-derived ubiquitin antibody. Flag antibodies were used to detect the protein levels of RIPK1, and OPTN was used to detect OPTN levels in whole-cell lysates. In the right panel, ImageJ software was used to semiquantitatively analyze ubiquitin levels. The data present means ± S.M. of independent experiment. OPTN silenced or overexpressed BV2 cells compared with control group, *P < 0.05, **P < 0.01

As discussed above, genetic interventions of OPTN have the ability to negatively regulate the protein accumulation of RIPK1 without affecting the mRNA expression of RIPK1 in BV2 cells (Fig. 6A, C, I, L, N, T). These results indicate that OPTN does not regulate RIPK1 at the transcriptional level. Given this, we speculated that the protein-degrading process, such as autophagy or the ubiquitin proteasome-degrading pathway, might be disrupted by OPTN intervention. To this end, we treated BV2 cells with either bafilomycin (Bafi) A1 to block the lysosomal pathway of autophagy or MG132 to inhibit the proteasome pathway in OPTN-overexpressing cells. Treatment with BafiA1 significantly decreased protein levels in OPTN-overexpressing BV2 cells (Fig. 8A, B). In contrast, MG132 treatment blocked the effects of OPTN on reducing protein levels of RIPK1 in BV2 cells (Fig. 8A, B). Therefore, the ubiquitin proteasome pathway seems to contribute to mediating the effects of OPTN on RIPK1 degradation in microglial cells.

To confirm the above findings, BV2 cells were transfected with Flag-RIPK1 in the absence or presence of OPTN knockdown by shRNAs. Immunoprecipitation with an anti-Flag antibody revealed the amount of ubiquitin that was bound, which was probed and visualized by western blots. The results demonstrated that knocking down the expression of OPTN markedly disrupted the binding between RIPK1 and ubiquitin in BV2 cells (Fig. 8C). Reciprocally, binding between RIPK1 and ubiquitin was strengthened by overexpressing RIPK1 in BV2 cells (Fig. 8D). Based on these observations, OPTN facilitates the ubiquitination of RIPK1 in microglial cells.

### The UBAN domain of OPTN and the death domain of RIPK1 mediate their interaction

Since optineurin (OPTN) is a ubiquitin-binding receptor protein [12, 64], we speculated that OPTN might be able to facilitate the ubiquitination of RIPK1 through a direct interaction. Using computational biology, we initially predicted the structures of OPTN and RIPK1. The C-score of RIPK1 was − 2.41, and the TM score was 0.43 ± 4.0 Å. For OPTN, the C-score was − 1.35, and the TM score was 0.55 ± 0.15 Å (Additional file 1: Fig. S1A). Based on their molecular structures, molecular docking was employed to predict the probability of binding between OPTN and RIPK1 using Haddock software [65]. The results demonstrated that the binding affinity, ΔG, was − 14.3 kcal/mol and that the dissociation constant Kd (M) at 25.0 °C was 3.1 × 10^–11^ (Additional file 1: Fig. S1B). Generally, the interaction is considered stable if Kd is less than 1 × 10^–9^. Therefore, OPTN may interact with RIPK1 according to the computational biology results. Analyzing the docking active amino acids, OPTN binds with RIPK1 through their N-termini and C-termini (Additional file 1: Fig. S1B).

**Fig. 9:**
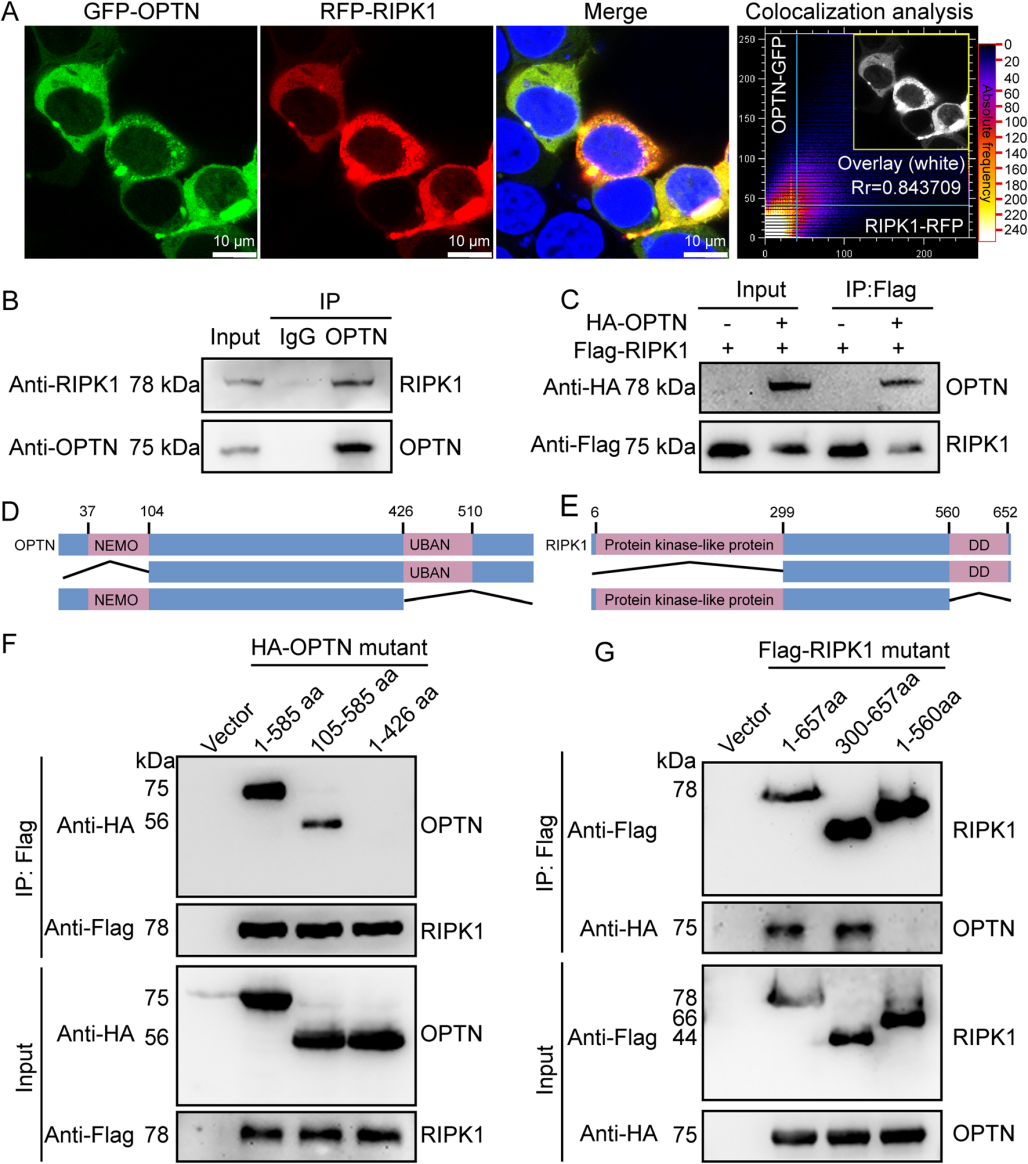
The UBAN domain of OPTN and the dead domain of RIPK1 mediate their interaction. **A** After 48 h of simultaneous overexpression of GFP-OPTN and mCherry-RIPK1 in BV2 cells, cells on the slide were fixed in PFA and stained with DAPI, followed by imaging using confocal microscopy. **B** The protein was precipitated using an OPTN antibody in BV2 cells and then detected using an RIPK1 antibody. **C** In HEK293T cells, both HA-OPTN and Flag-RIPK1 were overexpressed, which were then precipitated using an anti-Flag antibody and detected using an HA antibody. **D** Structural diagram of the OPTN protein. **E** Schematic diagram of RIPK1’s protein structure. **F** Full-length HA-OPTN, NEMO-truncated OPTN or UBAN-truncated OPTN were ectopically expressed in Flag-RIPK1-transfected HEK293T cells. Then, the proteins were immunoprecipitated using an anti-Flag antibody, followed by detection with an anti-HA antibody. **G** Full-length RIPK1, RIPK1 with a truncated protein-like kinase domain and RIPK1 with a truncated dead domain were ectopically expressed in HA-OPTN-transfected 293 T cells. Then, the proteins were immunoprecipitated using an anti-HA antibody, followed by detection with an anti-Flag antibody

To confirm the above prediction, we next overexpressed GFP-OPTN and mCherry-RIPK1 in BV2 cells. Using confocal microscopy, we found that OPTN colocalized with RIPK1 in BV2 cells (Fig. 9A). To further confirm their interaction, we immunoprecipitated OPTN using a specific antibody in BV2 cells. Using western blot, we found that RIPK1 co-immunoprecipitated with OPTN in BV2 cells, suggesting binding between OPTN and RIPK1 in microglial cells (Fig. 9B). In HEK293T cells, we cotransfected HA-OPTN and Flag-RIPK1. By immunoprecipitation using an anti-Flag antibody, we found that HA-OPTN co-immunoprecipitated with RIPK1, suggesting binding between OPTN and RIPK1 in HEK293T cells (Fig. 9C). From the results of the above immunoprecipitation, we fully confirmed the interaction between OPTN and RIPK1.

To determine the precise interacting domains, we initially analyzed the functional microdomains of OPTN and RIPK1. The results demonstrated that OPTN contains NEMO and UBAN domains, and RIPK1 contains protein kinase-like and death domains (Fig. 9D, E). According to this analysis, we established truncation fragments of HA-OPTN and Flag-RIPK1, which deleted the N- or C-terminal functional domains and were then cotransfected into HEK293T cells (Fig. 9F, G). By co-immunoprecipitation with either anti-Flag or anti-HA antibodies, we found that deletion of C-terminal functional domains disrupted the binding between OPTN and RIPK1 in HEK293T cells (Fig. 9F, G). Therefore, these results revealed that the UBAN domain of OPTN and the death domain of RIPK1 mediate their interaction in microglial cells.

### Restoration of OPTN decreases neuroinflammation by deactivating AIM2 inflammasomes and inducing RIPK1-degrading pathways in glial cells in APP/PS1 Tg mice

**Fig. 10:**
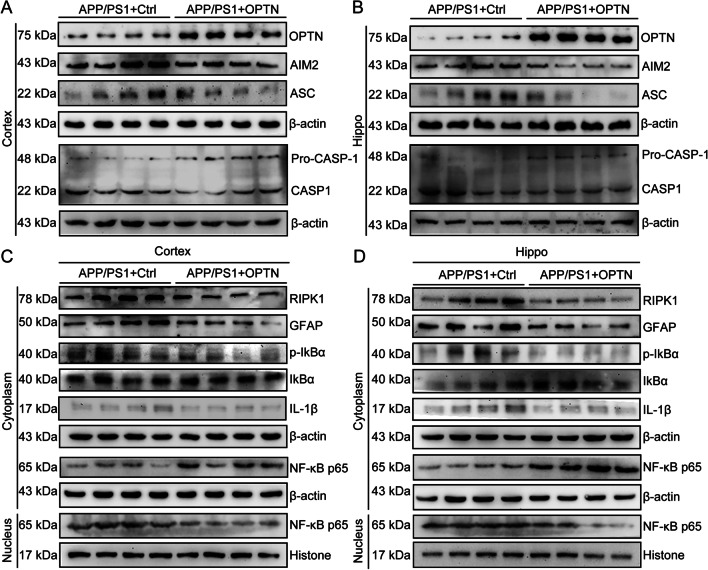
Overexpression of OPTN in the brains of APP/PS1 transgenic mice alleviates activation of the AIM2 inflammasome and RIPK1 pathways. **A**–**D** Three-month-old APP/PS1 transgenic mice were injected with OPTN or control adeno-associated virus in the hippocampus and cortex for 1 month. Brain tissue was collected after anesthesia euthanasia. **A**, **B** Western blotting was used to detect the protein expression of OPTN, AIM2, ASC and caspase-1 in the cerebral cortex and hippocampus of mice. β-actin served as the internal control. **C**, **D** Western blot analysis was used to determine levels of RIPK1, GFAP, p-IKBα, IKBα, IL-1β, and NF-κB in the cytoplasm and nucleus of mice. Histone and β-actin were used as internal controls for the nucleus and cytoplasm, respectively. The data present means ± S.M. of independent experiment. OPTN-AAV injected APP/PS1 Tg mice compared with control-AAV injected APP/PS1 Tg mice, *P < 0.05, **P < 0.01, ***P < 0.001

**Fig. 11:**
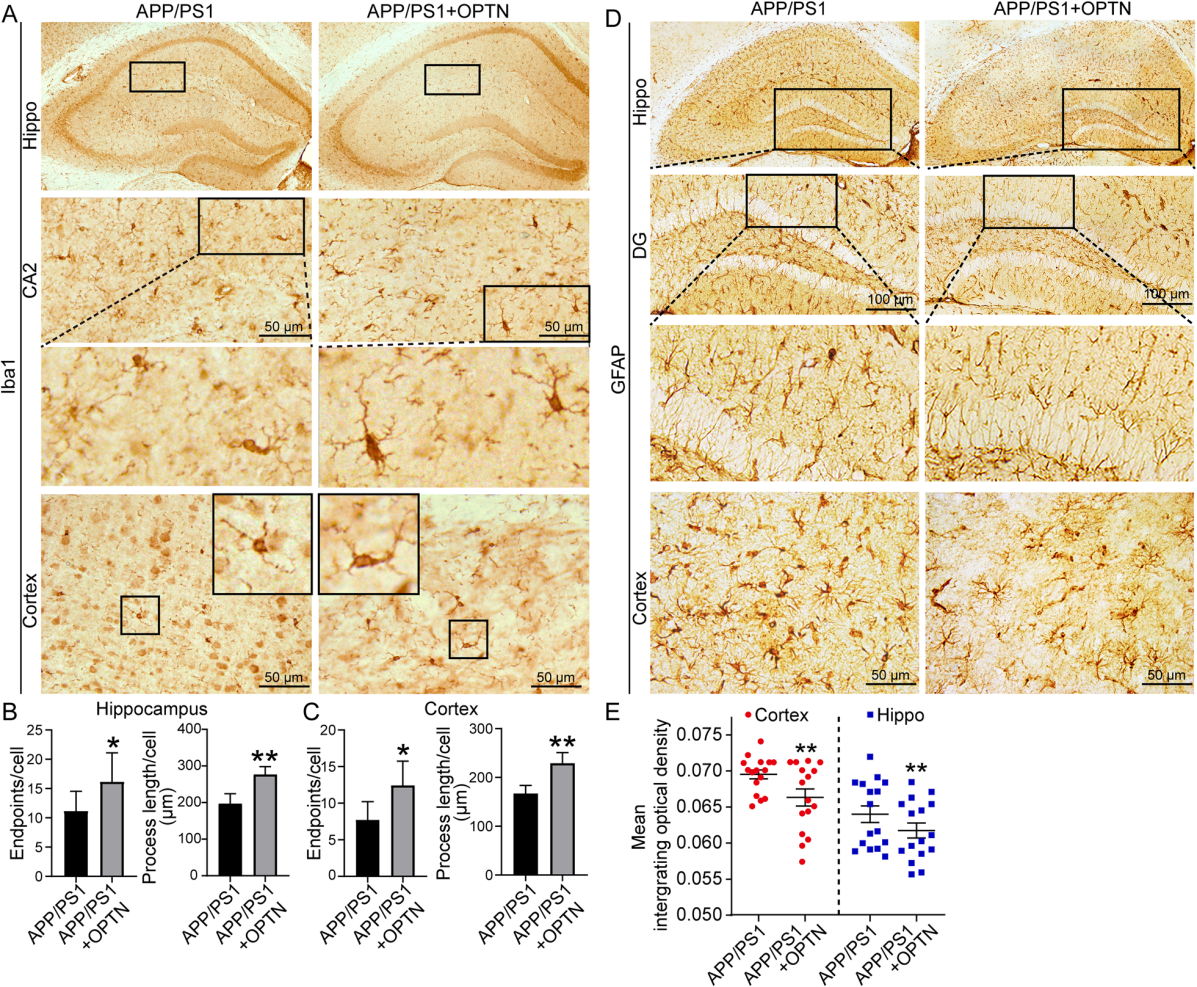
Overexpression of OPTN in the brains of APP/PS1 transgenic mice alleviates the activation of microglia and the number of astrocytes. **A**–**E** OPTN was ectopically overexpressed in the brains of 3-month-old APP/PS1 transgenic mice. **A** Iba1 immunohistochemical staining was performed in brain tissues of the OPTN-AAV-injected and control groups. **B**, **C** The activity of microglial cells was analyzed in hippocampal and cerebral cortex tissues. The data are presented as the means ± S.M. of independent experiment. **D** GFAP immunohistochemical staining was performed in the brains of the OPTN-overexpressing and control groups. **E** Statistical analysis of astrocytes. OPTN-AAV injected APP/PS1 Tg mice compared with control-AAV injected APP/PS1 Tg mcie, *P < 0.05, **P < 0.01

Because the expression of OPTN is downregulated in APP/PS1 Tg mice, we aimed to determine the effects of OPTN restoration on neuroinflammation. By injecting AAV-OPTN into the hippocampus of APP/PS1 Tg mice, we found that protein levels of AIM2, ASC, active forms of caspase-1 and the production of IL-1β were all repressed (Fig. 10A, B and Additional file 1: Fig. S2A–E). Similarly, OPTN overexpression decreased NF-κB translocation from the cytoplasm to the nucleus by reducing phosphorylation of IκBα in a RIPK1-dependent mechanism in APP/PS1 Tg mice (Fig. 10C, D and Additional file 1: Fig. S2F–L). By immunohistochemistry, Iba1 staining demonstrated that OPTN overexpression deactivates microglial cells by increasing their endpoints and process length (Fig. 11A–C). Additionally, the activity of astrocytes was also inhibited in response to ectopically expressed OPTN in APP/PS1 Tg mice (Fig. 11D, E). Based on these observations, OPTN is critical for suppressing neuroinflammation via the AIM2 inflammasome and RIPK-dependent NF-κB pathways.

## Discussion

AD is a progressive neurodegenerative disease that causes dementia. The primary symptoms associated with the disease are progressive loss of cognitive function and memory. The pathological mechanism of the disease is generally thought to be deposition of Aβ and hyperphosphorylation of tau [66]. Aβ deposition results in a potential innate immunopathological response in AD [67]. Indeed, the immune response is primarily focused on the deposition of Aβ and tangles of neuronal fibers [67, 68]. For example, APs are often closely associated with activated microglial cells and surrounded by activated astrocytes. Additional evidence suggests that cytokines, including IL-1β and IL-18, may contribute to the pathogenesis of AD [69]. Furthermore, studies have demonstrated that Aβ can activate the inflammasome, resulting in the secretion of IL-1β [21]. Treatment of AD model mice with inflammasome inhibitors significantly attenuated the decline in memory and reduced the deposition of APs [70]. In a subsequent study, APP/PS1/NLRP3^−/−^ mice exhibited reduced caspase-1 cleavage and Aβ deposition and enhanced phagocytosis of Aβ compared to APP/PS1 mice, providing evidence that NLRP3 plays an in vivo and exacerbating role in the pathogenesis of AD [45].

Based on these previous studies, we explored the activity of the AIM2 inflammasome in AD model mice. We found that the AIM2 inflammasome was also significantly activated, which was also confirmed in Aβ-treated microglial cells (Fig. 4). The AIM2 inflammasome was initially found to recognize and mediate the effects of pathogens [71] and host double-stranded DNA [72] on damaging the targeted tissues by inducing inflammation. Gradually, activation of the AIM2 inflammasome was found not to be restricted to only innate immune responses. In patients with type 2 diabetes, activation of the AIM2 inflammasome induced chronic inflammation [73]. In the brain, the AIM2 inflammasome contributes to neuroinflammation independently of NLRP3 [74], resulting in neuronal cell death [75]. Moreover, depletion of AIM2 in an AD mouse model mitigated the deposition of Aβ and microglial activation [76]. Our data revealed that the AIM2 inflammasome is activated in APP/PS1 Tg mice in response to the accumulation of Aβ in APs.

Similar to the NLRP3 inflammasome, the AIM2 inflammasome has also been identified as a multiprotein platform. Together with ASC and caspase-1, they induce the maturation of cytokines, such as IL-1β [40]. Consistently, we further found that production of the mature and active forms of caspase-1 and IL-1β was elevated in an Aβ-dependent manner (Fig. 4). In agreement with our observation, inflammatory factors, including caspase-1 and IL-1β, were upregulated following activation of the AIM2 inflammasome in patients infected with the hepatitis B virus [71]. Therefore, the AIM2 inflammasome is a potential essential mediator of neuroinflammation and a therapeutic target of AD.

With respect to activating AIM2 inflammasomes, OPTN deficiency was identified to be critical for this process in the brains of APP/PS1 Tg mice (Fig. 5). As a gene associated with normal tension glaucoma (NTG), OPTN was recently identified in NFTs and dystrophic neurites in AD patients, suggesting a new role for OPTN in the disease [77]. In addition to AD, OPTN was also identified to be expressed in the spinal cords of amyotrophic lateral sclerosis (ALS), Lewy bodies and Lewy neurites of Parkinson’s disease, ballooned neurons of Creutzfeldt–Jakob disease, glial cytoplasmic inclusions of multiple system atrophy, and Pick bodies of Pick’s disease, suggesting that OPTN may represent a more general marker for neurodegenerative diseases [78]. Although OPTN is widely distributed in neurodegenerative conditions, its significance is still obscure. Based on the above question, we initially extended prior works and demonstrated that the expression of OPTN was downregulated in the brains of AD patients and APP/PS1 Tg mice (Fig. 1A–G).

Given the above observation, we were prompted to determine OPTN’s roles in regulating the activity of AIM2 inflammasomes. As an essential receptor for mitochondrial autophagy [12], we found that OPTN restoration blocked the effects of Aβo on disrupting the fusion between mitochondria and lysosomes (Fig. 2). OPTN is recruited to the damaged outer mitochondrial membrane by binding to ubiquitinated mitochondrial proteins in a PTEN-induced putative kinase 1 (PINK1)- and parkin RBR E3 ubiquitin protein ligase (PARK2)-activating manner [79]. OPTN then induces the formation of autophagosomes around damaged mitochondria by interacting with the domain of the LC3 interaction region (LIR) [64]. Conversely, depletion of endogenous OPTN inhibits LC3 recruitment to mitochondria, resulting in inhibition of mitochondrial degradation [64]. As upstream modulators of LC3, autophagic factors, including unc-51-like autophagy activating kinase 1 (ULK1), FYVE-containing protein 1 (DFCP1) and WD-repeat protein interacting with phosphoinositides (WIPI) family 1 (WIPI1), are also recruited to focal regions proximal to the mitochondria [12]. Moreover, phosphorylation of OPTN by tank-binding kinase 1 (TBK1) at Ser473 enables its binding to the ubiquitin chains of pS65, which results in recruitment of OPTN to the mitochondria in PINK1-driven and parkin-independent mitophagy [80]. Although the biological functions of OPTN in mitophagy have been reported to be associated with neurodegenerative diseases, including ALS [64], Parkinson’s disease [79] and Huntington’s disease [81], the significance of OPTN in AD has not been elucidated. Therefore, we extended prior work on OPTN to AD, demonstrating that Aβ disrupts the fusion between impaired mitochondria and lysosomes by suppressing the expression of OPTN in microglial cells (Fig. 2B).

In response to the disruption of mitochondrial autophagy, impaired mitochondria release reactive oxygen species (ROS), free radicals and mitochondrial DNA (mtDNA) into the cytoplasm due to a lack of mitochondrial clearance, which may contribute to inflammation [59]. Indeed, AIM2 has been reported to bind to free cytoplasmic DNA through its HIN200 domain, which induces the oligomerization of ASC, leading to the formation of caspase-1-dependent inflammatory bodies and the maturation and secretion of proinflammatory cytokines, such as IL-1β and IL-18 [60]. Consistently, we further found that OPTN deficiency is critical for activating AIM2 inflammasomes in microglial cells (Fig. 5A).

We observed that OPTN deficiency induces accumulation of the RIPK1 protein in microglial cells (Fig. 8). In contrast to AIM2 inflammasomes, OPTN degrades RIPK1 via a proteasome pathway (Fig. 8). In agreement with our observation, RIPK1 is activated in response to proteasome inhibition [82]. In addition, there is evidence that RIPK1-mediated Cst7 induction leads to lysosomal pathway damage, which further induces the disease-associated microglia (DAM) phenotype, including an enhanced inflammatory response and decreased phagocytic activity [48]. However, the inherent mechanisms are not yet understood in microglial cells in the context of AD. To address this gap in knowledge, we extended prior works and found that OPTN recruits ubiquitinated RIPK1 to proteasomes by the UBAN domain of OPTN and the death domain of RIPK1 (Fig. 9F, G). These new findings provide a reasonable explanation for the fact that RIPK1 is activated by inhibiting the proteasome in microglial cells.

Apart from the regulatory mechanisms, we further found that ectopically expressed OPTN inhibited the proinflammatory pathways of NF-κB by degrading RIPK1 in APP/PS1 Tg mice (Fig. 10). RIPK1 mediates axonal degeneration by promoting inflammation and necroptosis in ALS [82]. As a key regulator of innate immune signaling pathways, heterozygous RIPK1 mutations prevent caspase-8 cleavage of RIPK1 in humans, which promotes autoinflammatory diseases [83]. With respect to the critical roles of RIPK1 in inflammation, RIPK1 has been identified as a therapeutic target of monogenic and polygenic autoimmune, inflammatory, neurodegenerative, ischemic and acute conditions, such as sepsis, by the potential applications of RIPK1 inhibitors [84]. Based on these clues, we extended prior works and demonstrated that RIPK1 mediates the effects of OPTN on suppressing neuroinflammation.

## Supplementary Information

**Additional file 1: Figure S1.** AIM2 knocking down is responsible for decreasing the cleavage of caspase1 and the production of IL-1β in microglial cells. **Figure S2.** OPTN was downregulated during the course of AD development and progression. **Figure S3.** OPTN was expressed in microglial cells of mice. **Figure S4.** The expression of NLRP3, NLRP1, Pyrin and NLRC4 in AD patients and APP/PS1 Tg mice. **Figure S5.** The mRNA expressions of OPTN were analyzed in the familial or sporadic AD patients.

## Data Availability

All data generated or analyzed during this study are included in this published article.

## Abbreviations

AAV: Adeno-associated virus
AD: Alzheimer’s disease
AIM2: Absent in melanoma 2
ALS: Amyotrophic lateral sclerosis
APs: β-Amyloid plaques
Aβ: β-Amyloid protein
Aβos: Aβ oligomers
Bafi: Bafilomycin
CARD: Contain a caspase recruitment domain
CoIP: Co-immunoprecipitation
DD: Death domain
FADD: FAS-associated death domain protein
GAPDH: Glyceraldehyde-3-phosphate dehydrogenase
GSDMD: Gasdermin-D
IHC: Immunohistochemistry
mtDNA: Mitochondrial DNA
NF-κB: Nuclear factor kappa B
NLRs: Nod-like receptors
NOD1: NLRs nucleotide-binding oligomerization domain-containing protein 1
OPTN: Optineurin
PET: Positron emission tomography
PYD: Pyrin domain
qRT-PCR: Quantitative real-time polymerase chain reaction
RAGE: Receptor for advanced glycation end products
RIPK1: Receptor interacting serine/threonine kinase 1
TLRs: Toll-like receptors
TNF-α: Tumor necrosis factor α
