# Supplementary material for: Downregulating expression of OPTN elevates neuroinflammation via AIM2 inflammasome- and RIPK1-activating mechanisms in APP/PS1 transgenic mice
Source: J Neuroinflammation. 2021 Dec 3;18:281. doi: 10.1186/s12974-021-02327-4 (PMC8641240; doi:10.1186/s12974-021-02327-4)
Supplement: Supplementary file 1 — Additional file 1: Figure S1. AIM2 knocking down is responsible for decreasing the cleavage of caspase1 and the production of IL-1β in microglial cells. Figure S2. OPTN was downregulated during the course of AD development and progression. Figure S3. OPTN was expressed in microglial cells of mice. Figure S4. The expression of NLRP3, NLRP1, Pyrin and NLRC4 in AD patients and APP/PS1 Tg mice. Figure S5. The mRNA expressions of OPTN were analyzed in the familial or sporadic AD patients. [file 12974_2021_2327_MOESM1_ESM.docx]

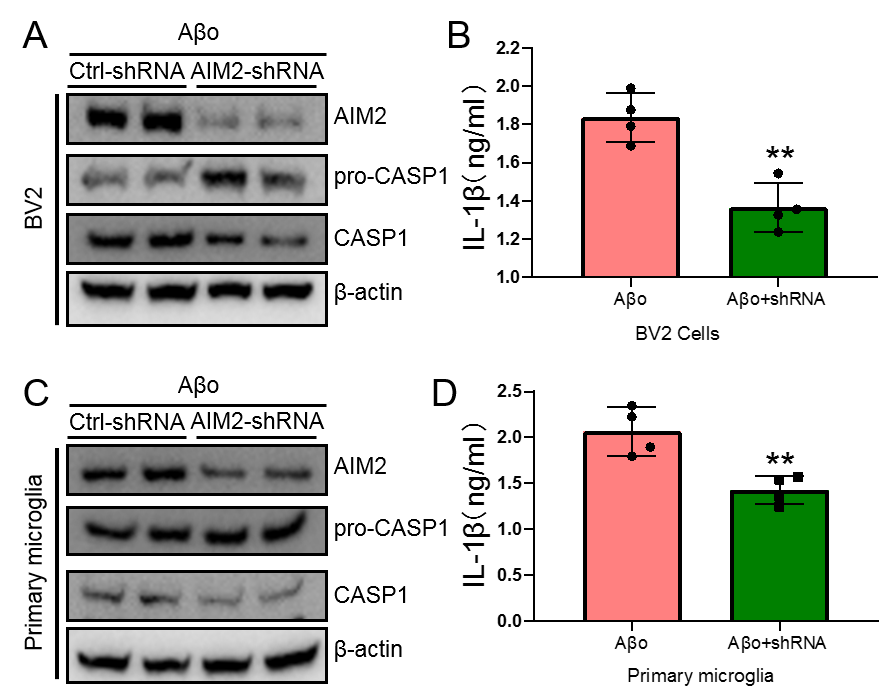


**Figure S1 to the reviewer’s comments. AIM2 knocking down is responsible for decreasing the cleavage of caspase1 and the production of IL-1β in microglial cells.** (A-D) AIM2 was knocked down by shRNA in BV2 or primary cultured microglial cells, which were further incubated with Aβo. (A, C) The protein levels of AIM2, pro-caspase1 and caspsae1 were determined by western blots. β-actin served as internal control. (B, D) The production of IL-1β was determined by ELISA. The data represent the means ± S.E. of independent experiments. AIM2 knocking down BV2 or primary cultured microglial cells were compared with non-interfering controls ** *P < 0.01*.


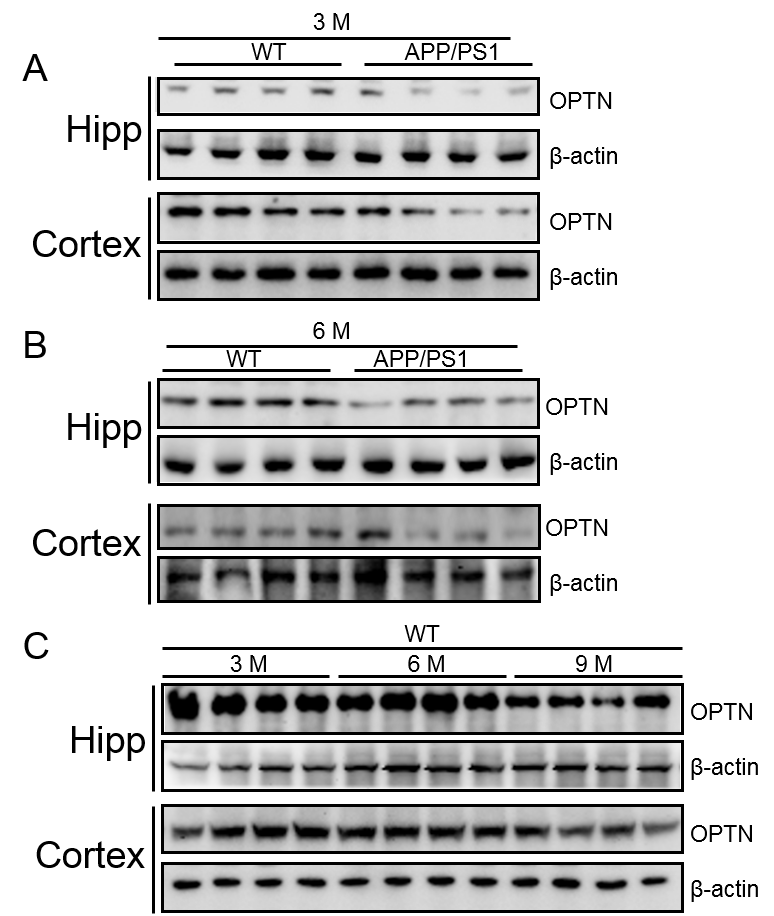


**Figure S2 to the reviewer’s comments. OPTN was downregulated during the course of AD development and progression.** Western blot was used to detect the expression of OPTN in hippocampus and cerebral cortex of WT and APP/PS1 transgenic mice at the age of (A) 3-month or (B) 6-month-old. (C) The protein levels of OPTN was determined by western blots in different age of C57BL/6 mice. β-actin served as internal control.


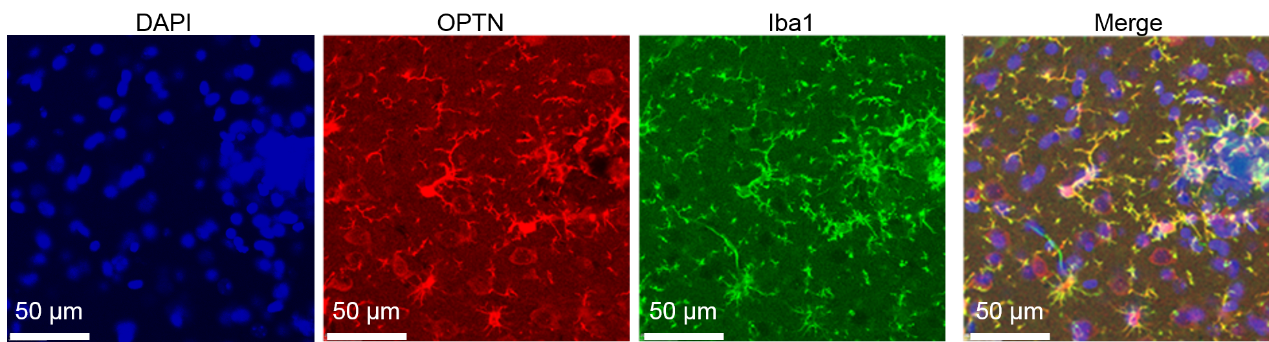


**Figure S3 to the reviewer’s comments. OPTN was expressed in microglial cells of mice.** The sections of were double-stained with OPTN (red) and Iba1 (Green). The merge image demonstrate the co-localization between OPTN and Iba1.


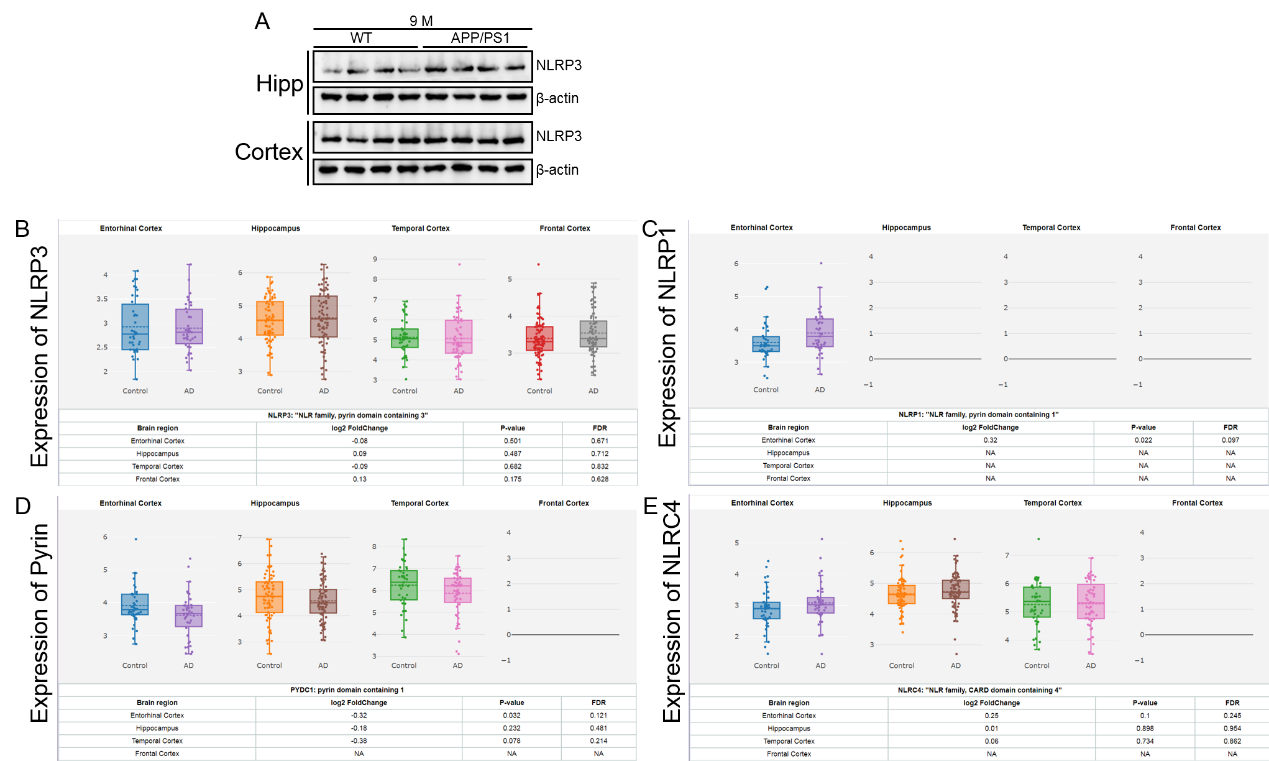


**Figure S4 to the reviewer’s comments. The expression of NLRP3, NLRP1, Pyrin and NLRC4 in AD patients and APP/PS1 Tg mice.** (A) Western blot was used to detect the expression of NLPR3 in the hippocampus and cerebral cortex of 9-month-old WT and APP/PS1 transgenic mice. β-actin served as internal control. (B-E) The mRNA expressions of NLRP3, NLRP1, Pyrin and NLRC4 were analyzed in GEO database.


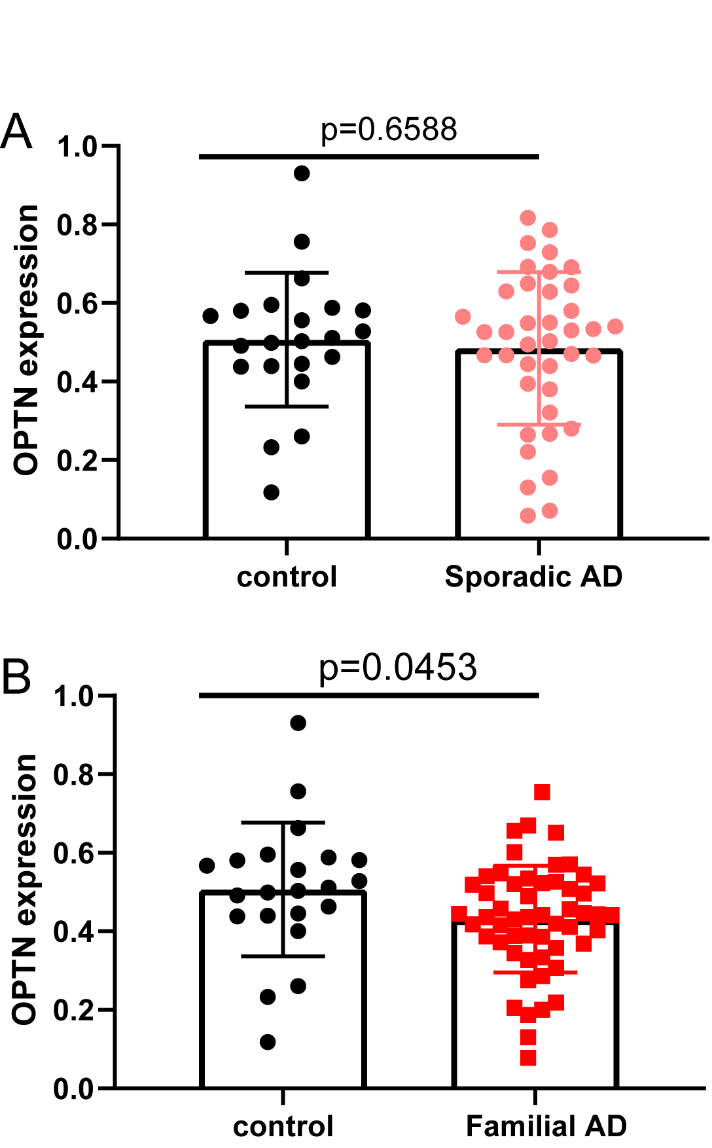


**Figure S5 to the reviewer’s comments. The mRNA expressions of OPTN were analyzed in the familial or sporadic AD patients.** (A) The mRNA expressions of OPTN were analyzed in sporadic AD patients. (B) The mRNA expression of OPTN was analyzed in familial AD patients.
